# Remotely supervised online cognitive training to reduce cognitive difficulties following chemotherapy in patients treated for localized breast cancer: Protocol of the Cog-Stim2 multicenter randomized controlled trial

**DOI:** 10.1371/journal.pone.0335124

**Published:** 2025-11-13

**Authors:** Marie Bousquet, Marie Lange, Justine Lequesne, Isabelle Durand-Zaleski, Olivia Diaz, Antonio Di Meglio, Thomas Bachelot, Jean-Michel Grellard, Bénédicte Clarisse, Florence Joly

**Affiliations:** 1 ANTICIPE U1086 INSERM-UCN, Equipe Labellisée Ligue Contre Le Cancer, Centre François Baclesse, Normandie Université UNICAEN, Caen, France; 2 Services Unit PLATON, Cancer and Cognition Platform, University of Caen Normandy, Caen, France; 3 Clinical Research Department, UNICANCER, Centre François Baclesse, Caen, France; 4 URC-ECO, Hôpital Hôtel-Dieu, AP-HP, Université de Paris, Paris, France; 5 Radiotherapy department, Daniel Hollard Institute, Grenoble, France; 6 Breast Cancer Unit, Medical Oncology Department, Gustave Roussy, Villejuif, France; 7 Department of Medical Oncology, Centre Leon Berard, Lyon, France; 8 Medical Oncology Department, CHU de Caen, Caen, France; PLOS: Public Library of Science, UNITED KINGDOM OF GREAT BRITAIN AND NORTHERN IRELAND

## Abstract

**Introduction:**

Chemotherapy-related cognitive impairment (CRCI) is frequently reported by breast cancer patients. Cognitive training is considered as one of the most effective approaches for improving cognitive function in patients with CRCI. As implementing cognitive training programs in healthcare centers remains challenging, online cognitive training appears to be an effective way to manage CRCI. Furthermore, supervision by a cognitive health specialist may increase motivation, adherence and effectiveness. However, the added benefit of combining supervision by health specialist with online cognitive training has not been studied in cancer patients.

**Method:**

Cog-Stim2 is a nationwide prospective multicenter randomized trial that aims to evaluate, in patients with localized breast cancer treated with chemotherapy who have cognitive complaints (n = 300), the benefits of a 12-week online cognitive training program (HappyNeuron®) supervised remotely by a neuropsychologist (experimental group) on cognitive complaints compared to open acess to the same cognitive training program for 12 weeks without supervision (active control group). Cognitive, associated factors, and biological assessments will be performed at baseline, at the end of the intervention, and 3 and 9 months later. The primary endpoint is the change in patients’ cognitive complaints (FACT‐Cog). The main secondary endpoints are objective cognitive functionning (CNS-VitalSign®), anxiety/depression (HADS), fatigue (FACIT-F) and sleep (ISI). The supervised sessions will be structured around patient’s difficulties and goals, and will include educational content (psychoeducation) on CRCI, the brain, cognition, and cognitive strategies to use in daily life. The medico-economic impact of the intervention will be also assessed.

**Discussion:**

The results will provide information on the additional benefits of combining remote supervision by experts with online cognitive training in a 12-week cognitive training program for breast cancer patients with CRCI. Our long-term goal is to generalize this type of intervention into clinical practice for patients with CRCI.

**Clinical trial registration:**

ClinicalTrials.gov identifier: NCT06027632, registered on 08/31/2023

## Introduction

Chemotherapy-related cognitive impairment (CRCI) is a frequently reported side effect (40–75%) in breast cancer patients [1,2]. It was previously known as ‘chemobrain’ due to its strong correlation with chemotherapy treatment. Symptoms often include difficulties in remembering, thinking, concentrating or finding the right words to express oneself, which can have a significant impact on patients’ quality of life and daily activities [2–4]. In addition, some patients may experience these symptoms for an extended period of time, up to 10 years after the end of treatments [5,6]. This can have a negative impact on the patient’s ability to return to work [2,7,8], and lead to a decrease of self-confidence in social situations. Thus, these difficulties can also have an economic impact on health insurers and patients’ workplaces, leading patients to take time off work or consult healthcare professionals more frequently without necessarily receiving appropriate help. It is therefore essential to implement interventions at the end of treatment to support patients as soon as cognitive symptoms appear and to prevent long-term CRCI.

It has been shown that a large majority (75%) of patients reporting CRCI are looking for support, particularly cognitive training, psychological support, and physical activity [9]. Furthermore, only a small number of supportive care departments offer CRCI interventions to patients [10], and healthcare providers often report not knowing how to respond to patients’ requests for CRCI support [11,12]. As a result, patients’ support needs often remain unmet.

Cognitive training is considered as one of the most effective approaches for improving cognitive function and quality of life in patients with CRCI [2,13]. Cognitive stimulation is a generic term that encompasses all activities aimed at improving cognition in general [14]. The two most common approaches are cognitive training and cognitive rehabilitation. Cognitive training is based on neuroplasticity and cognitive improvement through repeated and intensive practice. The exercises are generally adaptive, increasing in difficulty as the patient’s performance improves. The second approach, cognitive rehabilitation, may include cognitive training and psychoeducation. Psychoeducation includes educational content about the brain, cognition and cancer, and focuses on developing compensatory strategies to improve cognitive difficulties observed in daily life.

Despite their effectiveness, implementing cognitive stimulation programs in hospitals and care centers remains difficult. One of the main obstacles is the lack of specialists, such as neuropsychologists in oncology departments. Another challenge is the lack of adaptability of non-computerized interventions in hospitals to patients’ needs and schedules, which results in low adherence [15].

To overcome these limitations, emphasis has been placed on digital interventions. These interventions have been proven useful in healthcare and in improving mental health, as they can be delivered remotely using personal devices under the supervision of professionals [16–20]. Digital interventions are more accessible and affordable, particularly for isolated or stigmatized groups (such as patients living in rural areas and patients with special needs) and can be tailored to individual abilities. According to a systematic review [21], online cognitive training is an effective way to improve CRCI compared with standard care or other interventions in cancer patients. In particular, previous randomized trials have confirmed have confirmed the effectiveness of online cognitive training programs compared to standard care [22–26]. Overall, digital cognitive training has been shown to be an effective method for improving cognitive complaints [22,27] and should be considered the standard of care for the next generation of interventional studies. In addition, some studies have highlighted the importance of supervising interventions to ensure high levels of adherence [21,28,29]. It therefore seems essential that patients are supported by cognitive experts, even remotely, in order to maintain their motivation. Recently, European recommendations for CRCI interventions have been published, stipulating that effective interventions should include cognitive training and psychoeducation, preferably combined, under the supervision of cognition experts [15]. However, to date, the additional benefit of combining professional supervision, including psychoeducation, with online cognitive training has not been studied in patients with CRCI.

### Hypotheses and expected clinical outcomes

Our research hypothesis is that integrating personalized remote support supervised by a neuropsychologist into an online cognitive training program would enhance the effectiveness of the training on cognitive complaints. This would be achieved through improved participation/adherence to the online cognitive training program, as well as through the personalized supervision itself. We assume that supervision sessions, which include educational elements, will enable patients to identify their strengths, promote cognitive awareness, and develop individualized strategies for applying their compensatory skills in everyday life. Since CRCI has multiple underlying causes, reducing these symptoms requires a multifaceted approach. The combination of cognitive training (which improves neuroplasticity and directly targets the cognitive domains affected by cancer and its treatments) with structured supervised psychoeducation sessions based on compensatory strategies should reduce patients’ cognitive complaints more than online cognitive training alone.

In this context, we propose the first randomized comparative study to assess the effectiveness of a remotely supervised online cognitive training program, compared to an unsupervised online cognitive training program, in reducing cognitive complaints in patients with localized breast cancer after adjuvant chemotherapy. Our study aims to determine the additional benefits of remote supervision by a neuropsychologist to an online cognitive training, including psychoeducation sessions. The control group will have access to the same online cognitive training as the experimental group, but without any supervision.

## Methods

**Fig 1:**
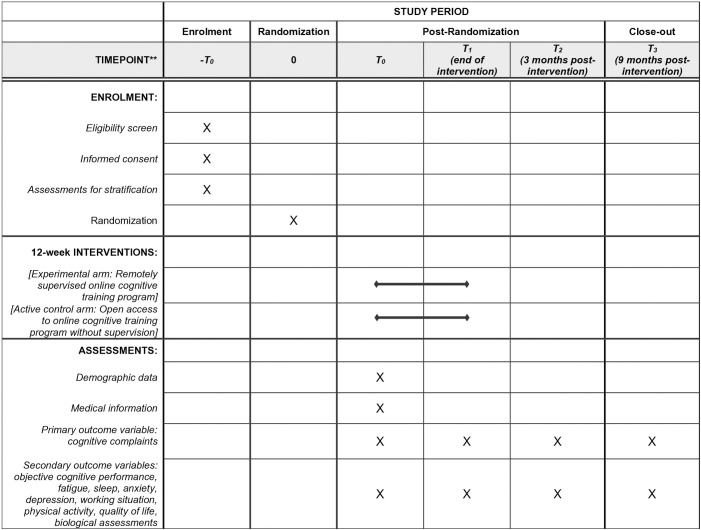
SPIRIT schedule of enrolment, interventions and assessment.

This study is a nationwide, open-label, multicenter, prospective, controlled, 1:1 randomized trial for patients with cognitive complaints following adjuvant chemotherapy for localized breast cancer. It compares a 12-week remotely supervised online cognitive training program with open access to online cognitive training without any supervision (Fig 1). We predict that patients who receive remote supervision by a neuropsychologist during their 12-week online cognitive training program will report a significant reduction in their cognitive complaints compared to those who only have access to the unsupervised online training at the end of the intervention. In addition, we plan to conduct follow-up assessments at 3 and 9 months after the end of the intervention to evaluate the delayed benefits of remote supervision. The Cog-Stim2 protocol and this manuscript were written in accordance with the Standard Protocol Items: Recommendations for Interventional Trials (SPIRIT) guidelines (S1 File).

This study has received ethical approval from the *Comité de Protection des Personnes Nord Ouest III* in August 2023 (N° ID RCB: 2023-A01134-41, Réf. SI RIPH 2G: 23.01946.000193). All patients will provide their informed written consent before the start of any study-related assessment. This study will be conducted in accordance with the relevant guidelines and regulations (Declaration of Helsinki).

### Objectives

The primary objective is to assess the change in cognitive complaints at the end of the program, from baseline, in both groups. The secondary objectives are to assess and compare the two groups of breast cancer patients based on various parameters:

- change of cognitive complaints during the follow-up (after the program),
- individual patient adherence to the online cognitive training program in each group,
- change in objective cognitive performances compared to baseline, at the end of the program and during follow-up,
- physical activity levels and health-related quality of life of patients,
- changes in patients’ fatigue, sleep, anxiety and depression from baseline, at the end of the program and during the follow-up,
- relationship between fatigue, sleep, anxiety, depression, physical activity and cognitive complaints/objective performances before the intervention, at the end of the program and during follow-up,
- proportion of patients who return to work among active patients at the end of the program and during follow-up,
- changes in biological parameters compared to baseline, at the end of the program and during follow-up,
- medico-economic impact of the intervention (see the “Medico-economic study” section).

### Study population

Patients must meet the following inclusion criteria: patients diagnosed with localized breast cancer, aged 18 years or older, who have received adjuvant or neoadjuvant chemotherapy and receiving adjuvant radiotherapy or until 6 months after the end of radiotherapy (ongoing hormonal therapy, CDK4/6 inhibitor, anti-HER2 treatment are permitted). Patients must report cognitive complaints that significantly affect their quality of life (assessed by the quality of life (QoL) subscale of the Functional Assessment of Cancer Therapy – Cognitive Function: FACT-Cog self-report questionnaire [30,31]). They must have completed at least three years of primary education, have access to a functional laptop/computer with a keyboard, internet connection and an email account – being able to use these tools independently, are fluent in French and have given informed consent to participate in the study.

Patients with one or more of the following non-inclusion criteria are not eligible: patients diagnosed with a personality disorder or any known progressive psychiatric pathology (e.g., schizophrenia), previous neurological history with persistent cognitive symptoms (head trauma, stroke, multiple sclerosis, epilepsy, neurodegenerative pathology, etc.), excessive alcohol or drug use, severe visual and/or hearing impairment, patient who may not be able to complete neuropsychological testing (including those with major cognitive disorders that interfere with the completion of cognitive tests, as determined by the cognitive screening test: Montreal Cognitive Assessment MoCA [32]), patients already participating in a cognitive training program, refusal to participate, patient deprived of their liberty or under guardianship, patients who may unable to participate for geographical, social or psychopathological reasons.

### Study sites

Seventeen centres are currently recruiting among the 25 French centers participating in the project. The list of study sites is available at https://clinicaltrials.gov/study/NCT06027632.

### Study experimental plan

**Fig 2:**
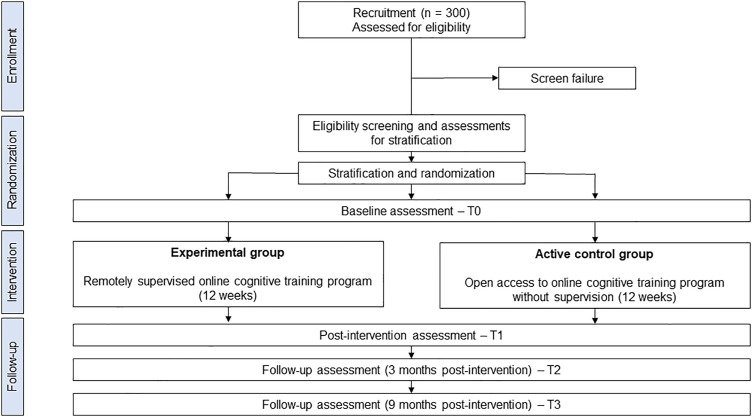
Study diagram.

Eligible patients who have signed informed consent form will be recruited. After baseline assessment, they will be randomized in a 1:1 ratio to be assigned to (Fig 2):

- the experimental group: 12-week of remotely supervised online cognitive training program,
- the active control group: 12-week of open access to online cognitive training without any supervision.

Randomization will be stratified according to: age (< 55 versus ≥ 55-years old), fatigue level (using the self-report questionnaire Functional Assessment of Chronic Illness Therapy‐Fatigue: FACIT-F [33]) [score: < 37 versus ≥ 37], anxiety and depression symptoms (using the the self-report questionnaire Hospital Anxiety and Depression Scale: HADS [34]) [scores: < 11 versus ≥ 11]; current physical activity (including the participation in an adapted physical activity (APA) program) using a visual analog scale: low (VAS 0–3), moderate (VAS 4–6), high (VAS 7–10)).

### The 12-week cognitive training program

#### Cognitive training – HappyNeuron Platform.

All patients will follow a 12-week online cognitive training program using the *HappyNeuron PRESCO®* online platform, with (experimental group) or without (active control group) remote supervision by a neuropsychologist. Cognitive training will begin within 3 weeks of inclusion.

The *HappyNeuron PRESCO®* software offers a standardized intervention targeting several cognitive domains that are impaired with CRCI: memory, executive functions, attention, information processing speed, language, ect. The cognitive training includes 38 exercises and 9 levels of difficulty, with an easy-to-use interface and a fun format to keep patients motivated. The difficulty level of the training will increase based on the patient’s performance. Patients will be notified by email three times a week that their 20-minute training session is ready. Patients in the experimental group will not have access to the platform outside of their scheduled training sessions, while patients in the active control group will have free access to the platform during the 12-week of the program.

#### Remote supervision of cognitive training.

Patients randomized to the experimental group will be contacted by a neuropsychologist via videoconference once a week for a 30-minute supervision session during the 12-week program. After an initial semi-structured interview aimed at identifying domains of cognitive complaints, each session will be structured around individual, professional, or personal development goals: the neuropsychologist will review patient’s difficulties during the week and discuss their strengths and vulnerabilities with them. Supervision sessions will include 15 minutes of psychoeducation on brain, cancer and cognition (CRCI, episodic memory, working memory, attention and processing speed, executive function), and the neuropsychologist will also suggest cognitive strategies to improve the patient’s performance, address difficulties encountered with the online training platform, and provide compensatory strategies to use in everyday life. This educational content has been developed by the team of neuropsychologists of the “Cancer and cognition” platform (Inserm U1086) at the Comprehensive Cancer Center of Caen (France), based on the methodology of Schuurs and Green [35] and the content of cognitive workshops already organized internally in the Oncology Supportive Care Department.

### Study assessments

All the participants will be assessed at inclusion – baseline (T0), at the end of the 12-week cognitive training program (T1), and then 3 (T2) and 9 months (T3) after the end of the intervention (Table 1). The project duration is estimated at 60 months, including 48 months of inclusion and 12 months of participation (12 weeks of cognitive intervention plus 9 months of follow-up) for each patient.

**Table 1:** Study flow-chart.

	Study information	Informed consent signature and eligibility	Assessment for stratification	RANDOMIZATION (ratio 1:1)	After randomization	Intervention (starting within 3 weeks after inclusion)	Follow-up after the end of the intervention		
			Up to 6 months after the end of radiotherapy		Weeks 1–12	At the end of the intervention	3 months after the end of the intervention	9 months after the end of the intervention
				**T0**		**T1**	**T2**	**T3**
Patient information and giving consent (15-day reflection period)	•							
*Informed Consent*		•			*Experimental arm: Remotely-supervised online cognitive training intervention* *Active control arm: Open access to online cognitive training without supervision*			
Medical information (cancer characteristics and treatments, co-morbidities…) Sociodemographic information				•				
Cognitive complaints: FACT-Cog: quality-of-life subscale (QoL) Full FACT-Cog		•		•		•	•	•
Overall cognitive abilities (MoCA test)		•						
Objective cognitive functions: CNS Vital Signs neuropsychological testing				•		•	•	•
Quality-of-life: FACT-G, EQ-5D-5L				•		•	•	•
Sleep: ISI self-report questionnaire				•		•	•	•
Fatigue: FACIT-F			•	•^1^		•	•	•
Anxiety/depression: HADS			•	•^1^		•	•	•
Physical activity (including APA): VAS IPAQ self-report questionnaire			•	•		•	•	•
Working situation				•		•	•	•
Satisfaction questionnaire						•		
Patient diary				•		•	•	
Biological assessments^2^				•		•	•	•
Biological sample collection *(optional)*					•		•		

^1^ *if baseline assessment more than 1 week after pre-randomization assessment.*

^2^Hematology (CBC, platelets, Hemoglobin), C-reactive protein (CRP), serum biochemistry (sodium, potassium, chloride, calcium, creatinine, glucose, ferritin), thyroid-function test (thyroid-stimulating hormone [TSH]).

#### Screening of eligible patients.

Patients will be screened based on their cognitive complaints and its impact on their quality of life, using the QoL subscale of the FACT-Cog questionnaire [30] based on normative data [31]); and based on the assessment of their cognitive status (using the MoCA cognitive screening test).

#### Pre-randomization assessments.

Patients wha have given their written informed consent will be assessed on site prior to randomization for the parameters considered for stratification:

- level of fatigue, using the FACIT-F [33],
- level of anxiety and depression, using the HADS [34],
- current physical activity (including participation in an adapted physical activity (APA) program), using a self-reported level of physical activity on a visual analog scale: low (VAS 0 to 3), moderate (VAS 4 to 6), high (VAS 7 to 10).

#### Assessments.

The study includes four assessment sessions: at baseline, before the start of the intervention (T0), at the end of the intervention (T1), then 3 months (T2) and 9 months (T3) after the end of the intervention. The assessments will include (Table 1):

- Medical information, such as cancer characteristics and treatments, as well as comorbidities from medical records, and sociodemographic information (age, education level, ect), at T0,
- Completion of Patient Reported Outcomes (PRO) self-report questionnaires to assess:
  - cognitive complaints, using the FACT-Cog [30], at T0, T1, T2, and T3,
  - quality of life, using the Functional Assessment of Cancer Therapy – General: FACT-G questionnaire [36] and the five-level measure of health status EQ-5D-5L [37], at T0, T1, T2, and T3,
  - sleep quality, using the self-report Insomnia Severity Index: ISI [38], at T0, T1, T2, and T3,
  - fatigue level, using the FACIT-F [33], and anxiety and depression level, using the HADS [34], at T0 (if more than one week after the pre-randomization assessment), T1, T2, and T3,
  - level of physical activity, using the International Physical Activity Questionnaire (IPAQ) [39], at T0, T1, T2, and T3,
- Assessment of objective cognitive functions, using the self-administered *CNS Vital Signs®* cognitive testing battery, at T0, T1, T2, and T3,
- A questionnaire assessing patient’s working situation (return to work, conditions of return to work: full or part-time, worplaces accomodations, professional retraining, ect), at T0, T1, T2, and T3,
- Biological assessments, at T0, T1, T2, and T3, including hematology (complete blood count, platelets, hemoglobin), C-reactive protein, serum biochemistry (sodium, potassium, chloride, calcium, creatinine, glucose, ferritin), and thyroid-function test (Thyroid-Stimulating Hormone (TSH)). An additional optional biological blood sample (6 tubes of 5 ml) will taken for future analysis at T0 and T1.
- At the end of the 12-week intervention (T1), patients will be asked to assess their satisfaction with the intervention using a questionnaire specific to each intervention group.

#### Medico-economic study.

We will conduct a within-study cost-utility analysis using standard methods, comparing online cognitive training supervised by a neuropsychologist with an unsupervised open-access online cognitive training program from a societal perspective. The results will be presented in terms of quality-adjusted life years (QALYs) and cumulative costs, not discounted due to the one-year time horizon (3 months intervention + 9 months follow-up). We will estimate the costs of all healthcare used in both groups, including:

- Labor costs for the supervised cognitive training,
- Physician services,
- Hospitalizations and emergency room visits,
- Outpatient diagnostic tests,
- Drugs, including the use of psychotropic medications,
- Home health care,
- Health-related out-of-pocket expenses and productivity costs.

The medico-economic evaluation will be based on national hospital admission costs, social health insurance service costs, and drug purchase prices. Productivity losses will be assessed by the mean gross domestic product (GDP) per capita. Healthcare costs will be compared between supervised and unsupervised groups for the period concerned, based on healthcare utilization data collected prospectively at the patient level using an electronic Case Report Form (eCRF) and hospital claims data. Overall quality of life assessed using the EQ-5D-5L will be used for the economic evaluation, to calculate QALYs. The cumulative costs and QALYs for each trial group will be estimated and compared in order to calculate the incremental cost-effectiveness ratio. Results will be expressed as incremental cost per QALYs. We will evaluate uncertainty and estimate confidence intervals around the estimates, using both deterministic and probabilistic methods. We will account for the correlation between costs and health outcomes using appropriate bivariate methods. Cost-effectiveness acceptability curves will be used to graphically represent the probability that the intervention is cost-effective for cost-effectiveness thresholds of €50 000 and €100 000 per QALY gained (multiple thresholds for sensitivity analyses). Reports will follow the Consolidated Health Economic Evaluation Reporting Standards.

## Statistical design overview

### Sample size calculation

This trial aims to assess the added value of remote supervision by a neuropsychologist of an online cognitive training, compared to an online cognitive training without any supervision. We anticipate a mean difference in cognitive complaints score change from baseline to the end of program (at 12 weeks) of 5.2 points or more on the continuous FACT-Cog Perceived Cognitive Impairment (PCI) between the experimental group and the control group (as observed in: Dos Santos *et al*., 2020 [27]). As previously suggested [40], this difference is clinically important. Using a two-sample t-test with equal variances (1:1 allocation ratio, bilateral alpha risk 0.05, power 80%) and assuming a standard deviation of 14.7, 127 assessable patients per group are required. To account for 15% of non-assessable patients, we plan to recruit 300 patients (150 per group).

### Statistical analyses

All analyses will be described in a detailed statistical analysis plan and will be performed on the intent-to-treat population. The continuous difference between FACT-Cog PCI subscale measurements at the end of the 12-week intervention and baseline measurements will be calculated and compared between experimental and control groups by using a bilateral Student’s t-test. PROs and objective cognitive assessments will be measured by scores and described as follows:

- Quantitative variables will be summarized using quartiles and ranges (mean and standard deviation for Gaussian variables),
- Categorical variables will be summarized using scores and proportions.

To measure changes over time, a comparison of scores between two assessments will first be performed by paired t-test (or the Wilcoxon signed-rank test for paired data for non-Gaussian variables) in each group. Next, a linear mixed model with random patient effect will be used to compare cognitive change between groups, adjusted for age, composite criteria of fatigue/anxiety/depression, and current physical activity (stratification criteria). For all statistical analyses, two-sided p-values <0.05 will be considered statistically significant.

### Quality control

#### Training of participating teams.

To ensure consistency across all participating centres, a comprehensive procedures manual will be prepared and approved by each centre prior to the start of the study. Study staff at each center will be trained on the consent process, eligibility screening, administering of the MoCA test (including training required for non-neuropsychologists), questionnaires, support for patients using the CNS vital Sign battery and the use of the HappyNeuron PRESCO platform.

#### Neuropsychological supervision.

In order to ensure consistent supervision, the neuropsychologist coordinator will thoroughly review a semi-structured interview questionnaire for the initial and follow-up interviews of patients in the experimental group, prior to the start of study. Weekly meetings will be held regurlarly to discuss patient progress and participation in the program, as required.

The educational content of these supervision sessions will be developed in advance and standardized among neuropsychologists involved in the project. This content will be based on the educational material already used by the neuropsychologists in the centre for cognitive workshops [35].

#### Protocol deviations.

A standard protocol deviations form will be used to track any deviations. The study staff will maintain the protocol deviations log. Study staff will also record deviations discovered during the data audit and reconcile errors.

#### Data monitoring and management.

The neuropsychologist coordinator will condust systematic monitoring of program adherence and patient improvements. Study staff will monitor data collection and protocol deviations. A data monitoring committee will not be necessary since the study is considered to be at minimal risk.

A Web-Based Data Capture (WBDC) system will be used for randomization, data collection and query handling. The investigator will ensure that data are recorded on the eCRFs as specified in the trial protocol and in accordance with the instructions provided. The investigator ensures the accuracy, completeness, and timeliness of the data recorded as well as of the provision of answers to data queries according to the Clinical Study Agreement. The investigator will be in charge of signature of the completed eCRFs. A copy of the completed eCRFs will be archived at the study site.

### Dissemination

Study results will be published in international peer-reviewed scientific journals and presented at relevant scientific conferences.

### Status and timeline of the study

This study is registered on ClinicalTrials.gov, identifier NCT06027632 and has been open for recruitment since February 9, 2024. Recruitment is due to finish in June 2028, and patient follow up complete in March 2029.

## Discussion

Due to the increasing proportion of survivors among breast cancer patients, scientific research must now focus on managing cancer- and treatment-related impairment. Furthermore, these patients require care and support to improve their quality of life and CRCI. However, implementing this type of support in hospitals and healthcare centers remains difficult for a variety of reasons: unavailability of specialists, cost of interventions, geographically isolated or stigmatized patients, lack of adaptability of non-computerized interventions, ect. With the Cog-Stim2 trial, we propose to study the added value of combining remote supervision by a neuropshychologist with online cognitive training as part of a 12-week support program for breast cancer patients with CRCI after adjuvant chemotherapy. Our long-term goal is to generalize this type of intervention in clinical practice for breast cancer patients with cognitive complaints and improve their quality of life. If our hypothesis is confirmed, this type of intervention could be widely implemented, reducing disparities in healthcare and potentially becoming a validated national cognitive remediation program for supportive care.

## Supporting information

S1 File SPIRIT checklist. (DOCX)

S2 File Copy of the study protocol, version 6.1 dated from 2025/03/17. (PDF)

S3 File Approval from the Comité de Protection des Personnes du Nord Ouest III (ethics committee). (DOCX)

S4 File Proof of external funding from French Health Ministry and French Cancer Institute (*Institut National du Cancer* (INCa)). (DOCX)
