# Supplementary material for: Remotely supervised online cognitive training to reduce cognitive difficulties following chemotherapy in patients treated for localized breast cancer: Protocol of the Cog-Stim2 multicenter randomized controlled trial
Source: PLoS One. 2025 Nov 13;20(11):e0335124. doi: 10.1371/journal.pone.0335124 (PMC12614541; doi:10.1371/journal.pone.0335124)
Supplement: S2 File — (PDF) [file pone.0335124.s002.pdf]

COG STIM 2 TRIAL

Remotely supervised computerized cognitive stimulation to reduce post-chemotherapy cognitive difficulties in patients treated for localized breast cancer: a multicenter randomized controlled trial

N° ID-RCB: 2023-A01134-41
Version 6.1 dated from 2025/03/17

This trial is financed within the framework of PHRC-Cancer 2022 (PHRC-K 22-038). It benefits from the scientific support of the French Breast Cancer Intergroup (UCBG), the Unicancer Translational Research and Development Group in Radiation Oncology (UNITRAD), and the French Unicancer Supportive care group (AFSOS).

REGLEMENTARY CLASSIFICATION OF THE TRIAL:

Recherche Interventionnelle impliquant la Personne Humaine de catégorie 2 (RIPH2)

**SPONSOR**	**Centre François Baclesse**<br>3 avenue du Général Harris<br>14076 CAEN cedex 5<br>Tél. : 02 31 45 50 50 – Fax : 02 31 45 51 58

**COORDONNATOR INVESTIGATOR**	**Pr Florence JOLY**<br>Centre François Baclesse<br>3 avenue du Général Harris<br>14076 CAEN cedex 5<br>E-mail : f.joly@baclesse.unicancer.fr<br>Tél. : 02 31 45 50 02 – Fax : 02 31 45 51 58

**ETHIC COMMITTEE**	CPP Nord-Ouest III	Date of approval : 16/08/2023<br>Amendement 1 autorisé le 7/10/2023<br>Amendement 2 autorisé le 13/06/2024<br>Amendement 3 autorisé le 6/07/2024<br>Amendement 4 autorisé le 7/12/2024

CONFIDENTIALITY STATEMENT

The information contained in this document is the property of Centre François Baclesse and therefore is provided to you in confidence for review by you, your team, an applicable Ethics Committee/Institutional Review and regulatory authorities. It is understood that the information will not be disclosed to third parties without prior written approval from Centre François Baclesse, except to the extent necessary to obtain informed consent from those persons to whom the medication may be administered.

PEOPLE INVOLVED IN THE PREPARATION AND THE CONDUCT OF THE PROTOCOL

**COORDINATING INVESTIGATOR**	Centre François Baclesse – CAEN<br>Tel : +33 (0)2 31 45 50 02<br>mail : f.joly@baclesse.unicancer.fr
**CLINICAL RESEARCH**<br><br>**Unit Leader**<br>Bénédicte CLARISSE<br><br>**Project Manager**<br>Jean-Michel GRELLARD<br><br>**Methodologist**<br>Justine LEQUESNE	Centre François Baclesse – CAEN<br>Tel : +33 (0)2 31 45 50 02<br>Fax : +33 (0)2 31 45 51 58<br><br>b.clarisse@baclesse.unicancer.fr<br><br>mail : jm.grellard@baclesse.unicancer.fr<br><br>mail : j.lequesne@baclesse.unicancer.fr
**HEALTH ECONOMICS**<br>Prof Isabelle DURAND-ZALESKI	Université de Paris, CRESS, INSERM, INRA, URCEco, AP-HP, Hôpital de l'Hôtel Dieu, F-75004, PARIS<br>Santé Publique Hôpital Henri Mondor<br>51 avenue du Maréchal de Lattre de Tassigny<br>F 94010 CRETEIL<br>Tel +33 (0)1 40 27 41 43<br>isabelle.durand-zaleski@aphp.fr
**COGNITION & QUALITY OF LIFE**<br>Marie LANGE<br>Marie BOUSQUET	Centre François Baclesse – CAEN<br>U1086 INSERM-UCBN - "Cancers & Préventions"<br>Tel : +33 (0)2 31 45 52 68<br>m.lange@baclesse.unicancer.fr<br><br>m.bousquet@baclesse.unicancer.fr

CONTENTS

1 SYNOPSIS 5

2 STUDY OVERVIEW 12

3 STUDY FLOW-CHART 13

4 SCIENTIFIC RATIONALE OF THE STUDY 14

4.1 BACKGROUND 14
4.2 HYPOTHESIS AND CLINICAL OUTCOMES 15

5 STUDY OBJECTIVES 16

5.1 PRIMARY OBJECTIVE 16
5.2 SECONDARY OBJECTIVES 16

6 ENDPOINTS 16

6.1 PRIMARY ENDPOINT 16
6.2 SECONDARY ENDPOINTS 16

7 STUDY DESIGN 17

7.1 METHODOLOGY 17
7.2 STUDY DURATION 18
7.3 SUBJECTS SELECTION 18
7.3.1 Inclusion criteria 18
7.3.2 Non-inclusion criteria 18
7.4 STUDY PLAN 18
7.4.1 Consent sign 18
7.4.2 Inclusion procedure 19
7.4.3 Intervention 19
7.4.4 Follow-up after the 12-week cognitive stimulation program (all patients) 20
7.5 SCHEDULE OF ASSESSMENTS 20
7.5.1 Screening of eligible patients with informed consent, prior to inclusion 20
7.5.2 Pre-randomization assessments (all patients) 20
7.5.3 Baseline assessments (within 3 weeks after-randomization, all patients) 20
7.5.4 Assessments during the 9-month follow-up after the end of the intervention 21
7.6 CRITERIA FOR PREMATURE WITHDRAWAL. 22

8 COGNITIVE EXERCISES 22

9 TOOLS FOR ASSESSMENTS 23

9.1 COGNITIVE COMPLAINTS: THE FACT-COG SELF-QUESTIONNAIRE 23
9.2 OBJECTIVE COGNITION FUNCTIONS: THE CNS VITAL SIGNS BATTERY 23
9.3 QUALITY OF-LIFE: THE FACT-G AND THE EQ-5D-5L SELF-QUESTIONNAIRES 24
9.4 QUALITY OF SLEEP: THE INSOMNIA SEVERITY INDEX (ISI) SELF-QUESTIONNAIRE 24
9.5 FATIGUE: THE FUNCTIONAL ASSESSMENT OF CHRONIC ILLNESS THERAPY FATIGUE (FACIT-F) SUBSCALE 24
9.6 ANXIETY/DEPRESSION SYMPTOMS: THE HOSPITAL ANXIETY AND DEPRESSION SCALE (HADS) 24
9.7 LEVEL OF PHYSICAL ACTIVITY: THE INTERNATIONAL PHYSICAL ACTIVITY QUESTIONNAIRE (IPAQ) 24
9.8 SOCIO-PROFESSIONAL CONDITIONS 24

10 SAFETY CONDUCTED AS CARE VIGILANCE 25

11 MEDICO-ECONOMIC STUDY 25

12 STATISTICAL CONSIDERATIONS 26

12.1 SAMPLE SIZE 26
12.2 STATISTICAL ANALYSIS 26
12.2.1 Primary objective 26
12.2.2 Secondary objectives 26

13 QUALITY CONTROL 27

13.1 TRAINING OF PARTICIPATING TEAMS 27
13.2 NEUROPSYCHOLOGICAL SUPERVISION 27

13.3 PROTOCOL DEVIATIONS 27
13.4 MONITORING 27

14 ETHICS AND REGULATORY CONSIDERATIONS 27

14.1 CLINICAL TRIAL AUTHORISATION 27
14.2 INFORMATION OF PATIENTS INVOLVED IN THE RESEARCH 28
14.3 INVESTIGATOR RESPONSIBILITIES 28
14.4 DATA CONFIDENTIALITY 29

15 DATA AND DOCUMENTS KEEPING 29

15.1 DATA ENTRY AND HANDLING 29
15.2 ARCHIVING 29
15.3 PUBLICATION POLICY 29

16 FUNDING AND INSURANCE 30

16.1 FUNDING 30
16.2 INSURANCE 30

17 REFERENCES 30

1 SYNOPSIS

**TITLE**	**Remotely supervised computerized cognitive stimulation to reduce post-chemotherapy cognitive difficulties in patients treated for localized breast cancer: a multicenter randomized controlled trial**
**ACRONYM**	**COG STIM2**
**Coordinator**	**Prof Florence JOLY, Medical Oncologist, Centre François Baclesse, Caen**
**Indication**	Patient with locally advanced breast cancer
**Objectives**	**Main objective**<br>To evaluate, in patients with localized breast cancer, the benefit of a 12-week computerized cognitive stimulation program supervised by a neuropsychologist (experimental group) on cognitive complaints compared to unsupervised 12-week open access to the same program (control group).<br>The evaluation of the benefit will be based on the change in cognitive complaints at the end of the program compared to baseline.<br>**Secondary objectives**<br>To evaluate and compare two groups of breast cancer patients on several parameters, including:<br>• *Individual adherence of patients to the online cognitive stimulation program in each group*<br>• *Change in cognitive complaints at T1 (end of intervention), T2 (3 months after the intervention) and T3 (9 months the intervention)*<br>• *Change in objective cognitive performances at T1, T2 and T3*<br>• *Health-related quality of life of patients at T1, T2 and T3*<br>• *Physical activity levels at T1, T2 and T3*<br>• *Changes of fatigue, sleep, anxiety and depression of patients at T1, T2 and T3*<br>• *The relationship between fatigue, sleep, anxiety, depression, physical activity and cognitive complaints/performances*<br>• *The proportion of patients who return to work at T1, T2 and T3 among working patients*<br>• *The medico-economic impact of the intervention*<br>• *The change in biological parameters*

<table>
<tr><td>Judgement criteria</td><td>
Main criterion
The primary endpoint is the average change in the score of the Perceived Cognitive Impairment (PCI) subscale score of the Functional Assessment of Cancer Therapy–Cognitive Function (FACT-Cog; cognitive complaints) after completion of the 12-weeks program, compared to the baseline score.
Secondary criteria
The secondary endpoints for both groups are as follows:
♦ Adherence rate to the program (proportion of patients who realized at least 24 out of 36 sessions planned for the entire program)
♦ At each time evaluation (baseline (T0), end of the program (T1), 3 months (T2) and 9 months (T3) after the end of the program), the following parameters will be assessed:
o Self-report cognitive complaint scores, including the PCI subscale and three other subscales of the FACT-Cog (perceived cognitive abilities, impact on quality of life, and comments from others).
o Scores of objective cognitive performances (based on the domains of attention, memory, executive functions and processing speed) assessed with the software CNS Vital Signs (CNS VS),
o Quality of life scores (FACT-G + EQ-5D-5L),
o Fatigue scores, evaluated with the self-report questionnaire Functional Assessment of Chronic Illness Therapy-Fatigue (FACIT-F),
o Sleep scores, evaluated with the self-report questionnaire Insomnia Severity Index (ISI),
o Anxiety and depression, evaluated with the self-report questionnaire Hospital Anxiety and Depression Scale (HADS),
o The level of physical activity will be evaluated using the International Physical Activity Questionnaire (IPAQ)
o Measure of the biological parameters
♦ The proportion of patients who return to work, the delay from randomization to date of return to work, and conditions of return to work (full or part-time, teleworking, professional retraining etc…),
♦ The cost of the intervention and the incremental cost-effectiveness ratio in € per Quality-Adjusted Life Year (QALY)
</td></tr>
<tr><td>Inclusion criteria</td><td>
- Patient diagnosed with localized breast cancer
- Age 18 or older,
- Who have received adjuvant or neo-adjuvant chemotherapy and are currently undergoing adjuvant radiotherapy (ongoing hormone therapy, maintenance therapy
</td></tr>
</table>

	other as target therapies or immunotherapy are permitted) until 6 months after end of radiotherapy.<br>- Patients who report cognitive complaints that significantly impact their quality of life, as evaluated by the quality of life subscale of the FACT-Cog questionnaire. This subscale is composed by 4 questions:<br>*1. I have been upset about these problems;*<br>*2. These problems have interfered with my ability to work;*<br>*3. These problems have interfered with my ability to do things I enjoy;*<br>*4. These problems have interfered with the quality of my life.*<br>Patients are eligible if their score on this subscale is at or below the 10th percentile, based on age guidelines and normative data (Lange et al., 2015), namely:<br>o ≤ 8 for patients aged 30-49 years<br>o ≤ 9 for patients aged 50-69 years<br>o ≤ 10 for patients aged 70-89 years<br>- Patients who have completed at least three years of primary school education, as determined by the Barbizet scale,<br>- Patient with access to a functional laptop/computer with a keyboard, internet connection and an e-mail account - being able to use those tools alone,<br>- Fluent in French,<br>- Patients who have provided informed consent to participate in the study.
**Non inclusion criteria**	- Personality disorder or any known progressive psychiatric pathology (e.g. schizophrenia),<br>- Previous neurological history with ongoing cognitive symptoms (sequelae of head trauma, stroke, multiple sclerosis, epilepsy, neurodegenerative pathology, etc.),<br>- Excessive alcohol intake or drug use, which could compromise participation to the intervention<br>- Major visual and/or hearing deficit,<br>- Patient who might not be able to complete neuropsychological testing, (including those with significant cognitive disorders that impede the completion of cognition tests, as determined by the cognitive screening test MoCA and based on age and educational level according to GRECOGVASC normative data)<br>- Already participating in a cognitive training program,<br>- Refusal to participate,<br>- Patient deprived of liberty or under guardianship,<br>- Patient who might not be able to participate due to geographic, social or psychopathological reasons.

**Experimental Plan**	**Prior to randomization**<br><br>Prior to randomization, verification of all eligibility criteria will be conducted, along with obtaining written informed consent. Additionally, participants will be required to fill out the following questionnaires to determine the composite criterion for stratification:<br>▪ Self-report questionnaire FACIT-F (fatigue)<br>▪ Self-report questionnaire HADS (anxiety/depression)<br>▪ Current physical activity (including the participation to a program of adapted physical activity (APA)), using a self-reported level of physical activity according to a visual analog scale: low (VAS 0 to 3), moderate (VAS 4 to 6), high (VAS 7 to 10)<br><br>**Randomization**<br><br>Enrolled localized breast cancer patients reporting cognitive complaints following chemotherapy will be randomized (1:1) between:<br>• ***Experimental arm:*** *Remotely-supervised online cognitive stimulation intervention*<br>• ***Active control arm:*** *Open access to online home-based cognitive exercises without supervision*<br><br>Randomisation will be stratified on:<br>- Age: <55 versus ≥55 year-old<br>- A composite criterion based on fatigue, anxiety, and depression (score <37 at the FACIT-F self-report questionnaire for evaluation of fatigue, and/or score > 11 at the HADS scale for evaluation of depression/anxiety, versus no abnormal score on either questionnaire) at inclusion<br>- Physical activity level at inclusion (low, moderate, high)<br><br>**Inclusion and baseline evaluation (T0) within 3 weeks after randomisation**<br><br>After inclusion, socio-demographic information and information on cancer characteristics, treatments and comorbidities will be collected.<br>A baseline assessment will be carried out **on-site** and will consist of the following:<br>▪ Self-report cognitive complaints: FACT-Cog self-report questionnaire<br>▪ Quality-of-life: FACT-G, EQ-5D-5L self-report questionnaires<br>▪ Sleep quality: ISI self-report questionnaire<br>▪ Fatigue*****: FACIT-F self-report questionnaire<br>▪ Anxiety/depression*****: HADS self-report questionnaire<br>▪ Physical activity level: IPAQ self-report questionnaire<br>▪ Working situation

	▪ Laboratory Assessments:<br>- Hematology (CBC, platelets, Hemoglobin)<br>- Serum biochemistry (Sodium, potassium, chloride, calcium, creatinine, glucose, ferritin),<br>- C-reactive protein (CRP)<br>- Thyroid-function testing: thyroid-stimulating hormone [TSH]<br>- Biological sample collection (optional)<br><br>***if baseline assessment more than 1 week after pre-randomization assessment***<br><br>Additionally, patients will complete the CNS Vital Signs neuropsychological testing battery to evaluate objective cognitive functions. This is a self-administered test on their personal computer **at home**, with instructions provided by a clinical research assistant via an information booklet prior to the test.<br><br>**Intervention**<br><br>All participants will have access to the "PRESCO" program of the "HAPPYNeuron-Pro" software, which is validated and designed to train up to 12 different cognitive domains, including attention, memory, executive functions and processing speed (Roussel-2016).<br><br>The experimental group will receive a 12-week intervention consisting of three 20-minute online cognitive stimulation sessions per week along with a weekly 30-minute centralized online remote supervision session with a neuropsychologist.<br>The neuropsychologist will support patients in the experimental arm during the supervision sessions (once per week). These sessions aim to identify patients' strengths, promoting their cognitive awareness, and developing individualized strategies to implement their compensatory abilities in real-life situations. They will be structured around individual professional or personal development goals, according to main cognitive impaired domains identified during an initial semi-structured interview. Moreover, each supervision session will include 15 minutes of educational session concerning:<br>- *Brain and cognition;*<br>- *Chemobrain;*<br>- *Episodic memory;*<br>- *Working memory, attention and processing speed;*<br>- *Executive functions;*<br>- *Recap of previous sessions and future steps*

The structure of the supervision intervention is based on Schuurs and Green approach (Schuurs and Green, 2013). The supervision will be centralized and provided remotely by neuropsychologists from the “Cancer and cognition” U1086 platform to ensure standardisation.

The intervention will involve a combination of online cognitive exercises and remote supervision based on two models for cognitive stimulation:

- *The restoration model, which suggests that repetitive performance-based training restores cognitive function by repairing neural networks whose function has been impaired (concept of neuroplasticity);*
- *The compensation model, which includes interventions to teach and reinforce new strategies for use in activities of daily living (concept of psychoeducation and metacognition).*

Assessments after the intervention

Evaluations will be performed **on-site:**

- at the end of the intervention (T1 = 3 months),
- 3 months (T2= 6 months) after the end of the intervention, and
- 9 months (T3= 12 months) after the end of the intervention

Each evaluation will include as follows:

- Cognitive complaints: FACT-Cog self-report questionnaire
- Quality-of-life: FACT-G, EQ-5D-5L
- Sleep quality: ISI self-report questionnaire
- Fatigue: FACIT-F self-report questionnaire
- Anxiety/depression: HADS self-report scale
- Physical activity level: IPAQ self-report questionnaire
- Return to work
- Laboratory Assessments:
    - Hematology (CBC, platelets, Hemoglobin)
    - Serum biochemistry (Sodium, potassium, chloride, calcium, creatinine, glucose, ferritine)
    - Thyroid-function testing: thyroid-stimulating hormone [TSH].
- Delivery of patient diary at T0, T1 and T2
- An optional biological sample collection will be proposed at the end of the intervention

Additionally, participants will complete the CNS Vital Signs neuropsychological testing battery to evaluate objective cognitive functions. This will be a self-administered test on their personal computer **at home**, with instructions provided by a clinical research assistant via an information booklet prior to the test.

**Participating centres**	127 assessable patients per arm are required. To anticipate 15% of non-assessable patients, we plan to enroll **<u>300 patients (150 per arm)</u>**
**Study duration**	Around 5 years, including:<br>- 48 months for inclusions<br>- 12 weeks of intervention<br>- 9 months of follow-up
**Medico-economic study**	A within-trial cost utility analysis using standard methods, comparing neuropsychologist-supervised home web-based cognitive stimulation to unsupervised home web-based cognitive stimulation from the societal perspective will be performed. Outcomes will be reported as quality-adjusted life years (QALYs) and cumulative costs, undiscounted due to the 1-year time horizon.

2 STUDY OVERVIEW

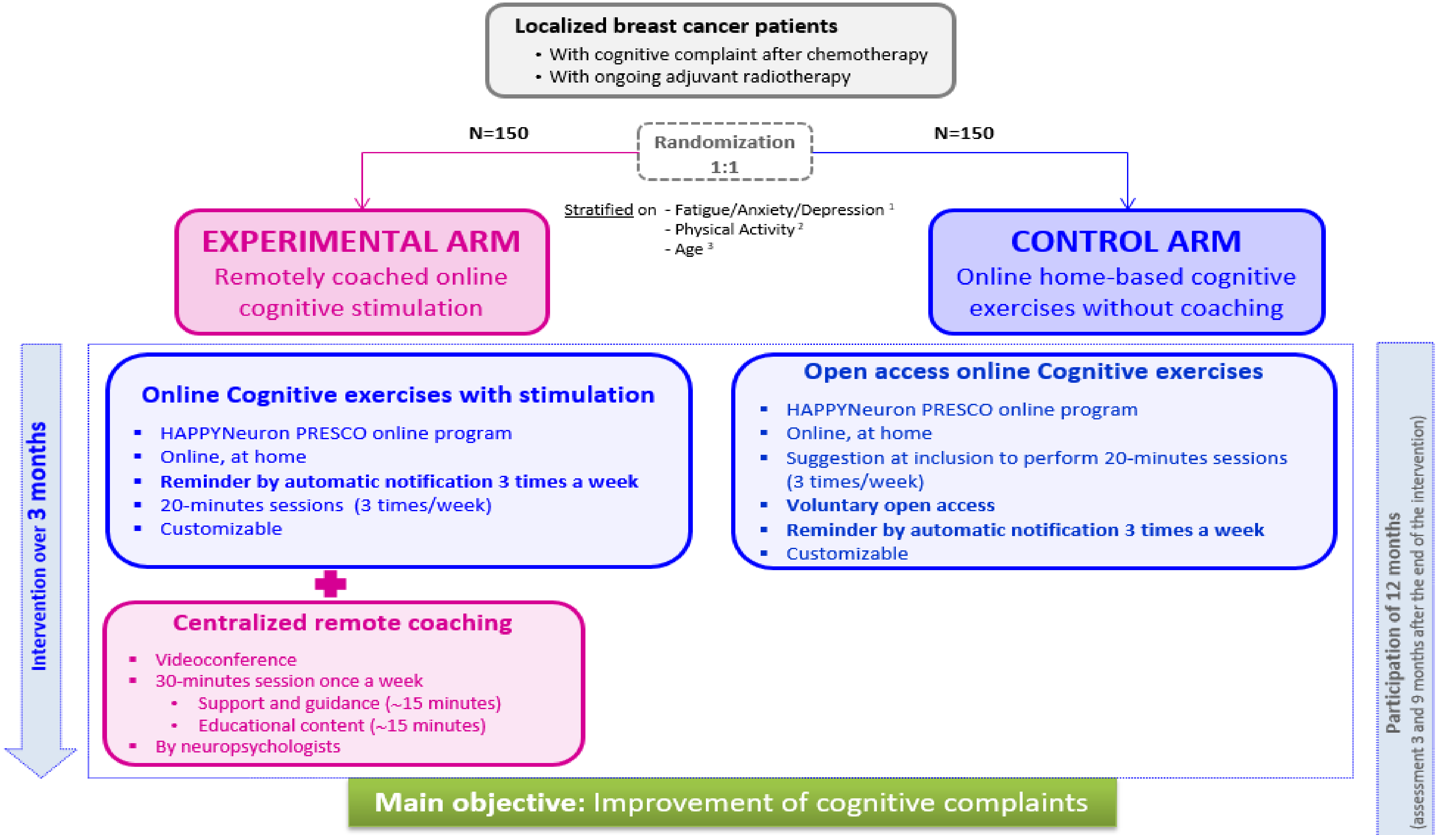

[1] Composite criteria taking into account fatigue, anxiety and depression: at least one impaired dimension (Fatigue (Fact-F score >37) and/or anxiety ($HADS_{anxiety}$ score >11) and/or depression ($HADS_{depression}$ score >11) ***versus*** none of them

[2] **Current Physical activity (including the participation to a program of adapted physical activity (APA))**, using a self-reported level of physical activity according to a visual analog scale (VAS): low (VAS 0 to 3), moderate (VAS 4 to 6), high (VAS 7 to 10)

[3] <55 ***versus*** ≥ 55 year-old

3 STUDY FLOW-CHART

	Study information	Informed consent signature and eligibility	Assessment for stratification		After randomization	Intervention (starting within 3 weeks after inclusion	Follow-up after the end of the intervention (if possible, in the same time of standard care visit; if not, a compensation of 30€ /visit will be proposed to the patient)		
			Up to 6 months after the end of radiotherapy			**Weeks 1 to 12**	At the end of the intervention	3 months after the end of the intervention	9 months after the end of the intervention
					**T0**		**T1**	**T2**	**T3**
**Patient information and giving consent** (15-day reflection period)	•			**RANDOMIZATION** (ratio 1:1)					
Informed Consent		•				***Experimental arm:*** *Remotely-supervised online cognitive stimulation intervention* ***Active control arm:*** *Open access to online home-based cognitive exercises without supervision*			
- **Medical information** (cancer characteristics and treatments, co-morbidities…)<br>- **Sociodemographic information**					•				
**Cognitive complaints**,<br>- FACT-Cog: quality-of-life subscale<br>- Full FACT-Cog		• (FACT-Cog: quality-of-life subscale)			• (Full FACT-Cog)		•	•	•
**General cognitive abilities** (MoCA screening test)		•							
**Objective cognitive functions**: CNS Vital Signs neuropsychological testing					•		•	•	•
**Quality-of-life**: FACT-G, EQ-5D-5L					•		•	•	•
**Sleep** : ISI self-report questionnaire					•		•	•	•
**Fatigue**: FACIT-F			•		•[1]		•	•	•
**Anxiety/depression:** HADS			•		•[1]		•	•	•
**Physical activity (including APA):**<br>- VAS<br>- IPAQ self-report questionnaire			• (VAS)		• (IPAQ)		•	•	•
**Working situation**					•		•	•	•
**Satisfaction questionnaire**							•		
**Patient diary**					•		•	•	
**Laboratory Assessments**[2]					•		•	•	•
**Biological sample collection** ***(optional)***					•		•		

[1] *if baseline assessment more than 1 week after pre-randomization assessment*

[2] - Hematology (CBC, platelets, Hemoglobin)
- Serum biochemistry (Sodium, potassium, chloride, calcium, creatinine, glucose, ferritine), CRP
- Thyroid-function testing: thyroid-stimulating hormone [TSH]

4 SCIENTIFIC RATIONALE OF THE STUDY

4.1 BACKGROUND

Cancer-related cognitive decline (CRCD) is a commonly reported side effect (40-75%) among breast cancer patients, which was previously referred to as chemobrain due to its strong correlation with chemotherapy treatments (Janelsins et al., 2017; Lange, Joly, et al., 2019) . Symptoms often include troubles in remembering, thinking, concentrating or finding the right words to express themselves, which can significantly impact patients' quality of life and daily activities (Joly et al., 2015; Lange, Joly, et al., 2019; Myers, 2013). Patients may experience these symptoms for an extended period, even up to 10 years after treatment (Koppelmans et al., 2012; Yamada et al., 2010). This can lead to a decrease in self-confidence at work and in social situations, as well as a negative impact on a patient's ability to return to work(Cheung et al., 2012; Munir et al., 2010; Nieuwenhuijsen et al., 2009; Von Ah et al., 2013). Therefore, it is crucial to provide interventions during treatment to support patients from the onset of symptoms and to prevent or delay the onset of CRCI for better long-term outcomes (Lonkhuizen et al., 2019).

Our survey of 1,600 patients revealed that a significant majority (75%) of those reporting CRCD expressed a strong desire for support, particularly in the form of cognitive stimulation, psychological assistance, and physical activity (Joly et al., 2015; Lange, Licaj, et al., 2019). Despite recommendations for CRCD interventions from various scientific organizations (NCNN, ASOS, ASCO, CNIB), there is currently no consensus on the most appropriate form of intervention. Furthermore, only a small number of supportive care departments provide intervention for CRCD to patients, and healthcare providers often report feeling uncertain about how to respond to patients' demand for CRCD support (Padgett et al., 2020; Smidt et al., 2016). Thus, patients' needs for support frequently remain unaddressed.

Cognitive stimulation is considered as one of the most effective approaches to enhance cognitive function and improve quality of life among patients with CRCD (Chan et al., 2015; Lange, Joly, et al., 2019). Cognitive stimulation is an umbrella term that covers all activities aiming at improving cognition in general (Clare & Woods, 2004). The two most common approaches are cognitive training, based on neuroplasticity and improvement through repeated and intensive exercises. The exercises are usually adaptive, and their difficulty increases as the patient's performance improves. The second is based on psychoeducation which focuses on developing compensatory strategies to improve overall cognitive resources. Given that CRCD has multiple causes, multimodal interventions may be the most appropriate strategy.

Despite their effectiveness, implementing cognitive stimulation programs in hospitals and care centres remains challenging. A major obstacle is the unavailability of professionals, such as neuropsychologists are in short supply and unable to meet the high demand from patients. Another challenge is the lack of adaptability of non-computerized interventions to the needs and schedules of patients, resulting in low adherence (Haberlin et al., 2018). Most patients are reluctant to return to the hospital after treatments for supportive cares.

To overcome these limits, the focus has shifted to digital interventions. These interventions have proven useful in the healthcare field and for improving mental health by allowing for remote delivery using personal devices under professional supervision (Barak et al., 2008; Luxton, 2015; Marks et al., 2007; Murray et al., 2004; Triberti et al., 2019). Digital interventions are more accessible and affordable, particularly for isolated or stigmatised groups (i.e. patients living in rural areas and patients with special needs) and can be tailored to individual abilities. They also provide standardized delivery, reducing proficiency bias (inequality in the application of the intervention due to differences between practitioners or resources at different sites). In addition, they may reduce the workload of healthcare professionals while minimizing errors.

According to a recent systematic review (Binarelli, Joly, et al., 2021), computerized cognitive stimulation is an effective way to improve CRCD, when compared to standard treatments or other interventions. For instance, the previous French randomized Cog-Reduc study (NCT01788618) showed that hospital software-based neuropsychologist-assisted cognitive stimulation reduced cognitive complaints, improved working memory, and resulted in better quality-of-life (Dos Santos et al., 2020). However, due to the format of the intervention (requiring the presence of a neuropsychologist and hospital-based program), it could not be widely implemented and was not adapted to all patients. Other studies in this review also had limitations, such as high risk of biases, no intention-to-treat analysis, non-blinded cognitive test assessors, missing outcome data,

and interventions mainly offered to cancer survivors long after the onset of CRCD, which typically begins during or after chemotherapy. Additionally, unsupervised interventions resulted in lower adherence.

Overall, digital cognitive stimulation has been shown to be effective in improving cognitive complaints and should be considered as standard reference for the next generation of interventional studies. It is also crucial to provide these interventions at home to make them more adapted to patients' and hospitals' schedules, especially during the COVID-19 pandemic. Furthermore, numerous studies have emphasized the importance of supervising the interventions to maintain high levels of adherence and compliance (Binarelli, Joly, et al., 2021; Kim & Kang, 2019). Hence, it appears essential for patients to be assisted by experts in cognition, even if remotely. However, to date, the added benefit of combining supervision including psycho-education strategies with online cognitive stimulation has not been investigated.

4.2 HYPOTHESIS AND CLINICAL OUTCOMES

We propose the first French randomized comparative study to assess the efficacy of a remotely supervised online cognitive stimulation program, compared to an unsupervised online cognitive exercise intervention, in reducing cognitive complaints in localized breast cancer patients after adjuvant chemotherapy. Previous randomized studies have confirmed the effectiveness of online cognitive stimulation programs compared to standard care (Bray et al., 2018; Conklin et al., 2015; Damholdt et al., 2016; Gehring et al., 2009; Mihuta et al., 2018). Our study seeks to determine the added value of remote supervision by a neuropsychologist. The control group will have access to the same online cognitive exercises as the experimental group but without supervision. We have chosen not to include a wait-list group as it would be unethical to deny patients with cognitive complaints the opportunity to participate in an intervention expected to benefit them. Our secondary objective is to evaluate the benefit of the supervised digitalized cognitive intervention on objective cognitive impairment.

Our research hypothesis is that incorporating personalized remote support with supervision from a neuropsychologist into a digitalized cognitive stimulation program will reinforce the effectiveness of the intervention on cognitive complaints. This will be achieved by improving participation/adherence to the online cognitive stimulation program, as well as through the personalized supervision itself. We believe that the supervision sessions, including educational components, will enable patients to identify their strengths, promote their cognitive awareness, and develop individualized strategies to apply their compensatory abilities in real-life situations. Since cognitive difficulties have multiple underlying causes, reducing these symptoms requires a multifaceted approach. Our hypothesis is that combining cognitive training (which increases neuroplasticity and directly targets the cognitive domains affected by cancer and its treatments), with structured supervised educational sessions based on compensatory strategies, will yield better outcomes than online cognitive stimulation alone.

The proposed combination of online cognitive exercises and remote supervision will rely on two models for cognitive stimulation:

- The restoration model, which suggests that repetitive performance-based training restores cognitive function by repairing neural networks whose function has been impaired (concept of neuroplasticity);
- The compensation model, which involves teaching and reinforcing new strategies for use in activities of daily living (concept of psychoeducation and metacognition).

Our proposed remotely support provided by a neuropsychologist will be structured with the following objectives:

- Maintaining patient motivation and interest in the program
- Addressing cognitive strengths and difficulties detected through the web-based intervention,
- Offering compensatory strategies,
- Identifying and discussing any difficulties patients experience while using the software,
- Detecting other possible factor that may influence the efficacy of the program (social and emotional aspects),
- Transferring the strategies learned during the program in everyday life.
- Educating patients on CRCI and cognition in general

It is worth noting that the supervision will be provided by neuropsychologists from the “Cancer and cognition” U1086 platform, which will increase standardisation and feasibility of the intervention.

We anticipate that patients who will receive remote neuropsychologist supervision during their 12-week digital cognitive stimulation program will report a significant decrease in cognitive complaints compared to those who only have access to the digital exercises without supervision. We expect a mean difference of 5.2 points in change of the FACT-Cog Perceived Cognitive Impairment (PCI) subscale between the two groups, based on

previous research we conducted with an on-site digital intervention (Dos Santos et al., 2020), which was found to have a clinically significant difference in outcomes (Bell et al., 2018).

In addition, we plan to conduct follow-up assessments at 3 and 9 months after the end of the intervention, to evaluate the continued benefit of remote supervision. This will also be taken into account in the planned cost-utility analysis.

The remote neuropsychologist-supervised intervention is well suited to the current COVID-19 pandemic, which has highlighted the importance of remote healthcare management for cancer patients. If our hypothesis is confirmed, this type of intervention could be widely implemented at a national level, reducing healthcare inequalities and potentially becoming a validated National Cognitive Remediation Program for cancer supportive care.

5 STUDY OBJECTIVES

5.1 PRIMARY OBJECTIVE

The primary objective is to evaluate, in patients with localized breast cancer, the benefit of a 12-week computerized cognitive stimulation program supervised by a neuropsychologist (experimental group) on cognitive complaints compared to unsupervised 12-week open access to the same program (control group). The evaluation of the benefit will be based on the change in cognitive complaints at the end of the program, as compared to baseline.

5.2 SECONDARY OBJECTIVES

The secondary objectives are to assess and compare the two groups of breast cancer patients based on various parameters:

- Individual adherence of patients to the online cognitive stimulation program in each group
- Change in cognitive complaints at T1 (end of intervention), T2 (3 months after the intervention) and T3 (9 months the intervention)
- Change of objective cognitive performances at T1, T2 and T3
- Health-related quality of life of patients at T1, T2 and T3
- Physical activity levels at T1, T2 and T3
- Changes of fatigue, sleep, anxiety and depression of patients at T1, T2 and T3
- The relationship between fatigue, sleep, anxiety, depression, physical activity and cognitive complaints/performances
- The proportion of patients who return to work at T1, T2 and T3 among working patients
- The change in biological parameters
- The medico-economic impact of the intervention (See the “Medico-economic study” section)

6 ENDPOINTS

6.1 PRIMARY ENDPOINT

The primary endpoint is the primary endpoint is the average change in the score of the Perceived Cognitive Impairment (PCI) subscale score of the Functional Assessment of Cancer Therapy–Cognitive Function (FACT-Cog) after completion of the 12-weeks program (T1), as compared to the baseline score.

6.2 SECONDARY ENDPOINTS

In both groups, the secondary endpoints are the following:

- Adherence rate to the program (proportion of patients who realized at least 24 out of 36 sessions planned for the entire program)
- At each time evaluation (baseline (T0), end of the program (T1), 3 months (T2) and 9 months (T3) after the end of the program), the following parameters will be assessed:
    - Self-report cognitive complaint scores, including the PCI subscale and three other subscales of the FACT-Cog (perceived cognitive abilities, impact on quality of life, and comments from others).

- Scores of objective cognitive domains (attention, memory, executive functions and processing speed) assessed with the software CNS Vital Signs (CNS VS),
  - Quality of life scores (FACT-G + EQ-5D-5L),
  - Fatigue scores, evaluated with the self-report questionnaire Functional Assessment of Chronic Illness Therapy-Fatigue (FACIT-F),
  - Sleep scores, evaluated with the self-report questionnaire Insomnia Severity Index (ISI),
  - Anxiety and depression, evaluated with the self-report questionnaire Hospital Anxiety and Depression Scale (HADS),
  - The level of physical activity will be evaluated using the International Physical Activity Questionnaire (IPAQ)
- The proportion of patients who return to work, the delay from randomization to date of return to work, and conditions of return to work (full or part-time, teleworking, professional retraining etc…),
- Measure of the biological parameters
- The Cost of the intervention and the incremental cost-effectiveness ratio in € per Quality-Adjusted Life Year (QALY)

7 STUDY DESIGN

7.1 METHODOLOGY

We propose a nationwide open-label multicentre prospective controlled randomized 1:1 trial for patients reporting cognitive complaints after adjuvant chemotherapy for localized breast cancer comparing a 12-weeks remotely supervised online cognitive stimulation program versus open access to online cognitive exercises without any supervision.

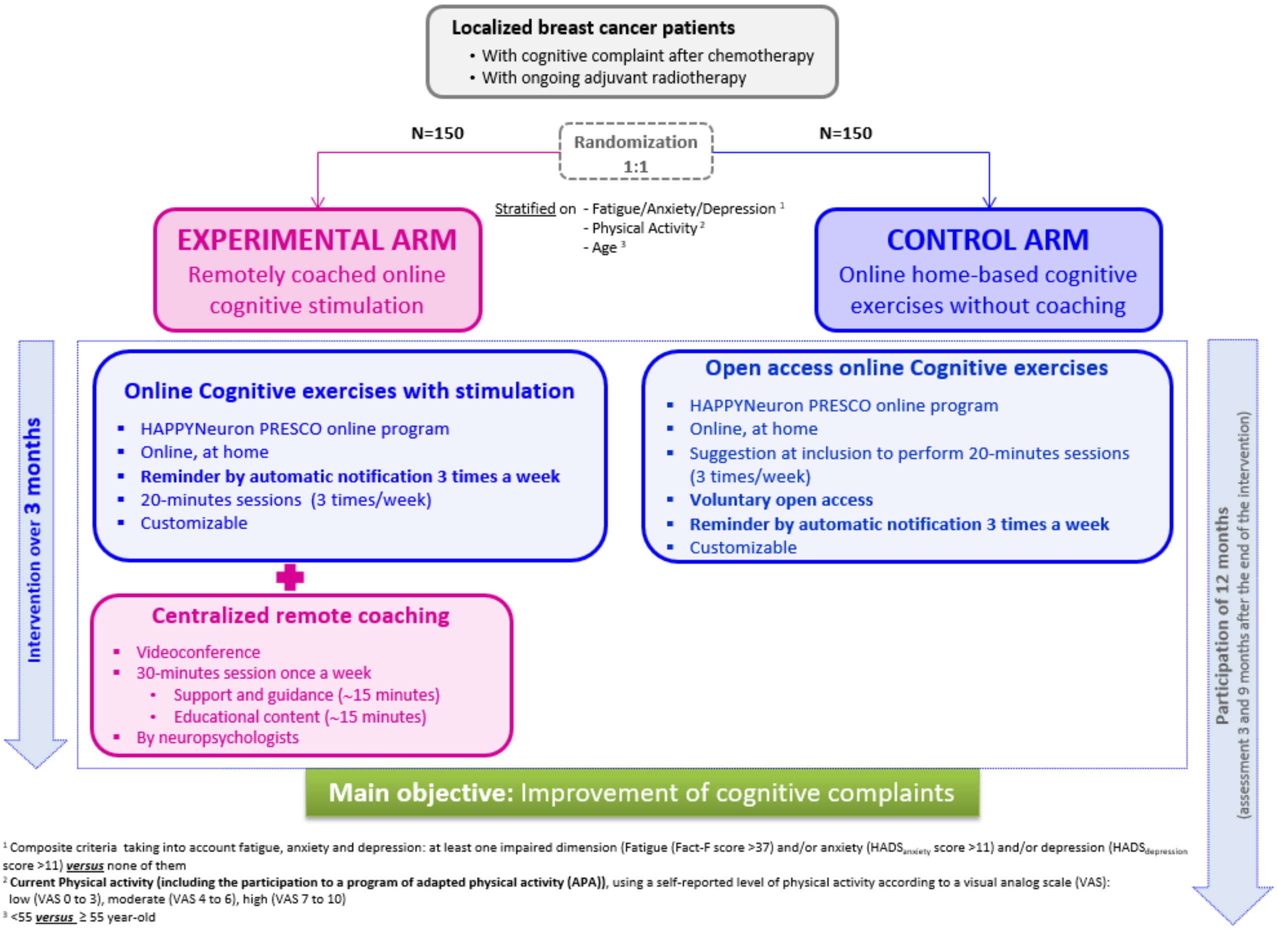

7.2 STUDY DURATION

The overall duration of the project is estimated as 60 months, including 48 months of inclusion and 12 months of participation (12 weeks of cognitive intervention plus 9 months of follow-up).

7.3 SUBJECTS SELECTION

7.3.1 Inclusion criteria

- Patients diagnosed with localized breast cancer
- Age 18 or older,
- Patients who have received adjuvant or neo-adjuvant chemotherapy and are currently undergoing adjuvant radiotherapy (ongoing hormone therapy, maintenance therapy other as target therapies or immunotherapies are permitted) until 6 months after end of radiotherapy.
- Patients who report cognitive complaints that significantly impact their quality of life, as evaluated by the quality of life subscale of the FACT-Cog questionnaire. This subscale is composed by 4 questions:
  1. *I have been upset about these problems;*
  2. *These problems have interfered with my ability to work;*
  3. *These problems have interfered with my ability to do things I enjoy;*
  4. *These problems have interfered with the quality of my life.*

  Patients are eligible if their score on this subscale is at or below the 10th percentile, based on age guidelines and normative data (Lange et al., 2015), namely:
    - ≤ 8 for patients aged 30-49 years
    - ≤ 9 for patients aged 50-69 years
    - ≤ 10 for patients aged 70-89 years
- Patients who have completed at least three years of primary school education, as determined by the Barbizet scale,
- Patient with access to a functional laptop/computer with a keyboard, internet connection and an e-mail account - being able to use those tools alone,
- Fluent in French,
- Patients who have provided informed consent to participate in the study.

7.3.2 Non-inclusion criteria

- Personality disorder or any known progressive psychiatric pathology (e.g. schizophrenia),
- Previous neurological history with ongoing cognitive symptoms (sequelae of head trauma, stroke, multiple sclerosis, epilepsy, neurodegenerative pathology, etc.),
- Excessive alcohol intake or drug use, which could compromise participation to the intervention
- Major visual and/or hearing deficit,
- Patient who might not be able to complete neuropsychological testing, (including those with significant cognitive disorders that impede the completion of cognition tests, as determined by the cognitive screening test MoCA and based on age and educational level according to GRECOGVASC normative data)
- Already participating in a cognitive training program,
- Refusal to participate,
- Patient deprived of liberty or under guardianship,
- Patients who might not be able to participate due to geographic, social or psychopathological reason

7.4 STUDY PLAN

7.4.1 Consent sign

Patients who meet the eligibility criteria will be presented with information about the study by the investigator. This will include an explanation of the study and provision of an information notice. Patients will be given ample time to consider their decision and reflect on the information provided before signing the informed consent.

Once the consent form is signed, the eligibility criteria will be reviewed to ensure that patients meet all eligibility criteria for the trial. The specific exams and procedures required for the baseline visit will be performed after the informed consent is obtained but before the patient is included in the trial.

7.4.2 Inclusion procedure

❖ *Screening process*

Patients with localized breast cancer reporting cognitive complaints after adjuvant chemotherapy and during breast radiotherapy will be informed about the study by their referring physician or the clinical research associate (CRA) at the investigator site. Patients will be given a 15-day period of reflection. Patients interested to participate to the study will sign the informed consent, after what they will then undergo a eligibility process to confirm their eligibility for inclusion and randomization.

This eligibility process will assess several parameters, including:

- the absence of major objective cognitive disorders, according to the score of the cognitive screening test MoCA, and
- the cognitive complaints impact on the quality of life of patient, assessed using the four questions from the QoL subscale of the self-report questionnaire FACT-Cog: eligibility will be confirmed if their score on this subscale is equal or below the 10th percentile, based on age guidelines and normative data (Lange et al., 2015).

❖ *Inclusion and randomization procedure*

Patients who meet all the eligibility criteria will be included in the study.

Before randomization takes place, patients will undergo, at the investigating center, an evaluation of various parameters required for stratification of the randomisation.

Randomisation will be stratified on:

- **Age**: <55 versus ≥55-year old,
- **Composite criterion** based on **fatigue, anxiety, and depression level**: score <37 at the FACIT-F self-report questionnaire for evaluation of fatigue, and/or score >11 at the HADS scale for evaluation of depression/anxiety versus no abnormal score at any of these questionnaires.
- **Physical activity level** (low, moderate, high, as assessed using a VAS)

Patients will then be randomized in a 1:1 ratio to be assigned to either:

- ***Experimental arm***: *12-weeks of remotely supervised online cognitive stimulation program*
- ***Active control arm***: *12-weeks of open access to online cognitive exercises without any supervision.*

7.4.3 Intervention

ALL patients (in experimental and active control groups)

❖ *All patients will follow a 12-weeks online cognitive exercises program, either with (experimental group) or without (active control group) remote neuropsychologist supervision. The cognitive stimulation will start within 3 weeks after inclusion.*

❖ *Patients in EXPERIMENTAL group*

Patients randomized in the **experimental arm** will first receive information concerning the online HappyNeuron PRESCO platform used for online cognitive exercises (see Section “Cognitive exercises” below for details) by the CRA of the investigator centre.

An initial interview through a 30-minute video-call will also be planned: it will be conducted by one of the neuropsychologists of the “Cancer and Cognition” (Inserm U1086) platform within **the Centre François Baclesse (Caen)**. This semi-structured interview will be based on information collected at baseline and will take place before the start of the cognitive stimulation program.

Thereafter, patients will:

- Receive reminders by automatic notification 3 times a week to complete three 20-minutes online cognitive exercises sessions using the HAPPYNeuron PRESCO online platform,
- Be contacted once a week for a 30-minute supervision session by a neuropsychologist via video-call. Each session will be structured around individual, professional or personal development goals, and the neuropsychologist will review patients’ performances of the week, and discuss with them their strengths and vulnerabilities. The neuropsychologist will also propose cognitive strategies to improve their performance, address any difficulties with the software and provide compensatory strategies to use in daily-life difficulties.

The supervision session will also include 15 minutes of educational content (psycho-education) concerning:

1. *Brain and cognition;*
2. *Chemobrain;*
3. *Episodic memory;*
4. *Working memory, attention and processing speed;*
5. *Executive functions;*
6. *Recap of previous sessions and future steps.*

This content was developed by the team of neuropsychologists from the "Cancer and cognition" (Inserm U1086) platform at the Centre François Baclesse, based on the methodology of Schuurs and Green (Schuurs and Green, 2013) and content from cognitive workshops already organized internally in the Oncology supportive care Department. It has also been proposed, in clinical practice for patients with CRCD and those planning to return to work (with the support of the French National League Against Cancer).

❖ *Patients in the ACTIVE CONTROL group*

Patients randomized in the **active control group** will first receive information concerning the online HappyNeuron PRESCO platform used for online cognitive exercises (see Section "Cognitive exercises" below for details) by the CRA of the investigator centre, who will in addition recommend to patients to perform at least 20 minutes of online cognitive exercises, 3 times per week.

Throughout the 12-week program, patients will

- Receive reminders by automatic notification 3 times a week to complete three 20-minutes online cognitive exercises sessions using the HAPPYNeuron PRESCO online platform,
- Have access to the cognitive exercises without any supervision or remote interviews with the neuropsychologists of the "Cancer and cognition" platform.

7.4.4 Follow-up after the 12-week cognitive stimulation program (all patients)

All patients from both groups will undergo a follow-up assessment at the end of the 12-week cognitive stimulation program (T1), thereafter at 3 (T2) and 9 months (T3), for a total participation period of approximately 12 months.

7.5 SCHEDULE OF ASSESSMENTS

7.5.1 Screening of eligible patients with informed consent, prior to inclusion

The following screening evaluations will be conducted on site for patients who express potential interest in participating to the study:

- Assessment of the degree of cognitive complaints, using the QoL subscale of the FACT-Cog self-report questionnaire
- Assessment of overall cognitive abilities, using the MoCA screening test
- Other eligibility criteria will be collected from medical records or through inquiries with patients during the screening evaluation

7.5.2 Pre-randomization assessments (all patients)

After patients have provided informed consent, and before randomization, the following parameters will be evaluated on site up to 6 months after the end of radiotherapy:

- **Fatigue level**, using the FACIT-F self-report questionnaire
- **Anxiety and depression level,** using the HADS self-report questionnaire
- **Current physical activity** (including the participation to a program of adapted physical activity (APA)), using a self-reported level of physical activity according to a visual analog scale: low (VAS 0 to 3), moderate (VAS 4 to 6), high (VAS 7 to 10)

7.5.3 Baseline assessments (within 3 weeks after-randomization, all patients)

Post-randomization, the on-site baseline evaluation (with estimated duration less than 1h) will include:

- The completion of self-administered questionnaires to assess:
    - **Cognitive complaints**, using the FACT-Cog self-report questionnaire
    - **Quality of life**, using the FACT-G and EQ-5D-5L self-report questionnaires

- **Sleep quality**, using the ISI self-report questionnaire
- **Fatigue level**, using the FACIT-F self-report questionnaire *(if baseline assessment more than 1 week after pre-randomization assessment)*
- **Anxiety/Depression level**, using the HADS self-report questionnaire *(if baseline assessment more than 1 week after pre-randomization assessment)*
- **Physical activity level**, using he IPAQ self-report questionnaire and reporting information concerning the participation to a program of adapted physical activity (APA)
- **Working situation**

- **Medical information,** such as cancer characteristics and treatments, as well as co-morbidities from medical records
- **Sociodemographic information** such as education level will be collected during the evaluation
- **Laboratory Assessments:**
  - Hematology (CBC, platelets, hemoglobin)
  - C-reactive protein
  - Serum biochemistry (Sodium, potassium, chloride, calcium, creatinine, glucose, ferritin)
  - Thyroid-function testing: thyroid-stimulating hormone [TSH]
  - Collection and conservation of blood samples for future explorations: A biological collection will be set up for future investigations from a blood sample (6 x 5 ml tubes). This collection is optional.
- All patient will also receive a patient diary at T0
- Additionally, participants will then complete: **The CNS Vital Signs neuropsychological testing battery** to evaluate objective cognitive functions. This will be a self-administered test on their personal computer at home, with instructions provided by a clinical research assistant via an information booklet prior to the test. Patients will not have access to their performance in these cognitive tests.

7.5.4 Assessments during the 9-month follow-up after the end of the intervention

Patients from both groups will undergo a follow-up assessment, realized similarly three times:

- within one week after completing the 12-week online cognitive stimulation program (T1)
- 3 months (T2) after the end of the program
- and 9 months (T3) after the end of the program

These follow-up assessments will be realized on site, and will be planned, if possible, in the same time of a standard care visit. In the case the patient will have to come on site for a protocol-specific follow-up assessment, a compensation of 30€ /visit will be proposed to the patient)

Evaluations will include, on site:

- The completion of self-administered questionnaires to assess:
  - **Cognitive complaints**, using the FACT-Cog self-report questionnaire
  - **Quality of life**, using the FACT-G and EQ-5D-5L self-report questionnaires
  - **Sleep quality**, using the ISI self-report questionnaire
  - **Fatigue level**, using the FACIT-F self-report questionnaire
  - **Anxiety/Depression level**, using the HADS self-report questionnaire
  - **Physical activity level**, using he IPAQ self-report questionnaire
  - **Return to work**: date of return to work, and conditions of return to work (full or part-time, teleworking, professional retraining…)
- **Delivery of patient diary at T0, T1 and T2**
- **Laboratory Assessments similarly as at baseline at the end of the programme, then 3 and 9 months after the end of the cognitive stimulation programme.:**
  - Hematology (CBC, platelets, hemoglobin)
  - C-reactive protein
  - Serum biochemistry (Sodium, potassium, chloride, calcium, creatinine, glucose, ferritin)
  - Thyroid-function testing: thyroid-stimulating hormone [TSH]

At the end of the 12-week intervention only (T1)**,** a new optional biological collection will be set up for future investigations from a blood sample (6 x 5 ml tubes).

Additionally, participants will complete:

- **The CNS Vital Signs neuropsychological testing battery** to evaluate objective cognitive functions. This will be a self-administered test on their personal computer at home, with instructions provided by a clinical research assistant via an information booklet prior to the test.

- **At the end of the 12-week intervention only** (T1)**,** patients will be asked to assess their satisfaction with regard to the intervention using a specific questionnaire for each modality of intervention.

7.6 CRITERIA FOR PREMATURE WITHDRAWAL.

The intervention will be discontinued in the following scenarios:

- Patient's decision (previously collected data can still be used unless the patient objects)
- Inter-current illness or other reasons that require stopping the participation to the study
- Patient becoming untraceable
- Investigator's decision

In the event that a patient chooses to withdraw from the intervention program early, they will still be monitored unless they withdraw from the study altogether.

8 COGNITIVE EXERCISES

Both groups of patients will use the online version of the **HAPPYNeuron PRESCO** platform for **cognitive exercises**. This program was developed by the French neurologist B. Croisile, in French, and is available in 11 languages allowing a comparison of results between international studies. It has been used in several clinical studies, including with breast cancer patients (Damholdt et al., 2016). It has also already been used by the sponsor team in a feasibility study with patients treated with radiotherapy for a breast cancer(Binarelli, Lange, et al., 2021), and a feasibility study with patients over 70 years old and in the cognitive workshops promoted at the centre for all patients reporting cognitive difficulties. In all cases, patients provided positive feedbacks concerning the use of the software. This program has been specifically developed to train, through its 41 ludic exercises and 9 levels of difficulty, a wide range of cognitive domains (12 in total) including the most impaired after cancer treatments: attention, memory, executive functions and processing speed (Joly et al., 2015; Lange, Licaj, et al., 2019). Due to its broad range of targeted cognitive domains and intuitive user interface, it is well-suited for this clinical study. A table detailing the cognitive exercises available to participating patients is provided below.

**Cognitive domains trained**	**Exercises**	
**Memory (verbal and visual)**	Words, Where are you?<br>Elephant Memory<br>Shapes and Colours<br>Heraldry<br>Displaced Characters<br>Displaced Images<br>N-Back	Around the World in 80 trips<br>I Remember You!<br>Restaurant<br>An American in Paris<br>Find Your Way!<br>Chunking<br>Objects, Where are You?
**Executive functions**	Towers of Hanoi<br>Basketball in New-York	Hurry for Change!
**Attention**	Pay Attention!<br>Private Eye!	Ancient Writing
**Information processing speed**	Under Pressure	Gulf Stream<br>Catch the Ladybug!
**Language**	Split words<br>Embroidery<br>Secret files<br>Speak Your Mind!	Decipher<br>Writing in the Stars<br>This Story is Full of Blanks!<br>Which One is Alike?
**Logic**	The Right Count	Ready, Steady, Count!

	Countdown	
**visuospatial abilities**	Sleight of Hands<br>Entangled Figures	Point of View<br>Turn Around and Around

9 TOOLS FOR ASSESSMENTS

9.1 COGNITIVE COMPLAINTS: THE FACT-COG SELF-QUESTIONNAIRE

After inclusion, patients' **cognitive complaints** will be evaluated using the self-report questionnaire **FACT-Cog**. It is a standardized and validated measure developed through interviews with expert clinicians and cancer patient focus groups (Joly et al., 2012; Wagner et al., 2009) and is widely used in clinical practice for evaluating patients' cognitive complaints during and after chemotherapy. It consists of four subscales:

1. *Perceived Cognitive Impairment (PCI, score range 0-72)*
2. *Perceived cognitive abilities (PCA score range 0-28)*
3. *Impact on quality of life (QOL score range 0-16)*
4. *Comments from others (OTH score range 0-16)*

Each item requires the patient to indicate on a 5-point Likert scale how frequently a particular situation has occurred in the past seven days.

9.2 OBJECTIVE COGNITION FUNCTIONS: THE CNS VITAL SIGNS BATTERY

The **CNS Vital Signs (CNS VS)** battery will be used to measure subtle cognitive performances differences inter and intra-group before and after intervention. This allow for easy cognitive assessment, without needing the presence of a neuropsychologist, as the test will be self-administered on a personal computer at home, with instructions provided by a clinical research assistant via an information booklet prior to the test. In addition, patients will be sent an email containing a link to access the test, a password, and guidelines to finish the 30-minute evaluation. The battery includes tests commonly used in neuropsychology, such as symbol digit coding, the Stroop test and finger tapping. Its test-retest reliability, concurrent validity with traditional tests, and discriminant validity were evaluated and found to be similar to those of traditional neuropsychological tests. The battery consists of 7 main tests that take approximately 30 minutes to complete.

- Verbal Memory (VBM): VBM measures recognition memory for Words. Fifteen words are presented, one by one, on the screen every two seconds. For immediate recognition, the participant has to identify those words nested among fifteen new words. Then, after six more tests, there is a delayed recognition trial.
- Visual Memory (VIM): VIM measures recognition memory for Figures or Shapes. Fifteen geometric figures are presented, one by one, on the screen. For immediate recognition, the participant has to identify those figures nested among fifteen new figures. Then, after five more tests, there is a delayed recognition trial.
- Finger Tapping (FTT): FTT test requires subjects to press the Space Bar with their right index finger as many times as they can in 10 seconds. They do this once for practice, and then there are three test trials. The test is repeated with the left hand.
- Symbol Digit Coding (SDC): SDC test consists of serial presentations of screens, each of which contains a bank of eight symbols above and eight empty boxes below. The participant types in the number that corresponds to the symbol that is highlighted. The computer program does not allow a person to use a numerical pad preventing a distinct advantage for those who are skilled at using the numerical pad or for those that are right- versus left-handed.
- Stroop Test (ST): In the first part, the words RED, YELLOW, BLUE, and GREEN (printed in black) appear at random on the screen, and the participant presses the space bar as soon as the test subject sees the word. In the second part, the words RED, YELLOW, BLUE, and GREEN appear on the screen, printed in colour. The participant is asked to press the space bar when the colour of the word matches what the word says. In the third part, the words RED, YELLOW, BLUE, and GREEN appear on the screen, printed in colour. The participant is asked to press the space bar when the colour of the word does not match what the word says.
- Shifting Attention (SAT): SAT test is a measure of ability to shift from one instruction set to another quickly and accurately. Participants are instructed to match geometric objects either by shape or by colour. Three figures appear on the screen, one on top and two on the bottom. The top figure is either a square or a circle. The bottom figures are a square and a circle. The figures are either red or blue (mixed randomly).

The participant is asked to match one of the bottom figures to the top figure. The rules change at random (i.e. match the figures by shape, for another, by colour).

- Continuous Performance (CPT): CPT test is a measure of vigilance or sustained attention. The test subject is asked to respond to the target stimulus “B” but not to any other letter. The stimuli are presented at random.

9.3 QUALITY OF-LIFE: THE FACT-G AND THE EQ-5D-5L SELF-QUESTIONNAIRES

- *FACT-G self-report questionnaire*

Quality of life will be evaluated by using the **FACT-G** self-report questionnaire (D. F. Cella et al., 1993), which is a 27-item compilation of general questions divided into four primary QOL domains: Physical Well-Being, Social/Family Well-Being, Emotional Well-Being, and Functional Well-Being. It is considered appropriate for use with patients with any form of cancer, and has also been used and validated in the general population (using a slightly modified version).

- *EQ-5D-5L self-report questionnaire*

In addition, quality of life will be also assessed through the French version of the five-level version of **EQ-5D** self-questionnaire (EQ-5D-5L), notably for the purpose of health-economics analysis. The EQ-5D is a generic instrument for describing and valuing health. It is based on a descriptive system that defines health in terms of 5 dimensions: Mobility, Self-Care, Usual Activities, Pain/Discomfort, and Anxiety/Depression (Brooks, 1996).

9.4 QUALITY OF SLEEP: THE INSOMNIA SEVERITY INDEX (ISI) SELF-QUESTIONNAIRE

Quality of sleep will be evaluated through the **Insomnia Severity Index (ISI).** This 7-item self-report questionnaire was designed to assess the severity, underlying factors and impact of insomnia and to monitor treatment response in adults (Morin et al., 2011). It allows to evaluate 6 domains of insomnia: Severity of sleep onset, sleep maintenance and early morning wakening problems, sleep dissatisfaction, interference of sleep difficulties with daytime functioning, noticeability of sleep problems by others, distress caused by the sleep difficulties.

9.5 FATIGUE: THE FUNCTIONAL ASSESSMENT OF CHRONIC ILLNESS THERAPY FATIGUE (FACIT-F) SUBSCALE

Fatigue will be evaluated by the completion of the **FACIT-F subscale.** This 13-item self-report questionnaire assesses self-reported fatigue and its impact on daily activities and function. It was originally developed as an addition to the Functional Assessment of Cancer Therapy (FACT) measurement system (D. Cella et al., 2002).

9.6 ANXIETY/DEPRESSION SYMPTOMS: THE HOSPITAL ANXIETY AND DEPRESSION SCALE (HADS)

Level of anxiety and depression will be evaluated by the **HADS.** This 14-items self-report questionnaire was originally developed by Zigmond and Snaith (1983) (Zigmond & Snaith, 1983) and is commonly used in the clinical practice to screen level of anxiety and depression in patients with various medical conditions.

9.7 LEVEL OF PHYSICAL ACTIVITY: THE INTERNATIONAL PHYSICAL ACTIVITY QUESTIONNAIRE (IPAQ)

Level of physical activity will be screened using the **IPAQ** (Craig et al., 2003). This standardized open-source self-report questionnaire provides an estimation of physical activity (intense, moderate and walking activities) and sedentary behaviour (sitting time) for adults across a range of socio-economic settings. The IPAQ short-form assesses the last 7-days physical activity undertaken across a comprehensive set of domains including: a. leisure time physical activity b. domestic and gardening (yard) activities c. work-related physical activity d. transport-related physical activity. The specific types of assessed activities are walking, moderate-intensity activities and vigorous-intensity activities.

9.8 SOCIO-PROFESSIONAL CONDITIONS

Socio-professional conditions will be assessed with a home-made questionnaire assessing notably date of return to work, and conditions of return to work (full or part-time, teleworking, professional retraining…)

10 SAFETY conducted as care vigilance

In accordance with the new research regulation involving the human person, there will be no collection of serious adverse events organized by the sponsor as part of the study.

However, as for all research involving the human person, the sponsor will transmit to the investigators concerned any information likely to affect the safety of persons (Art R1123-52 CSP) and will inform without delay the competent authority and the protection committee of new safety facts and, where appropriate, measures taken (Article R1123-59 of the CSP).

Nevertheless, health professionals are reminded that care vigilance applies, as a result any incident or adverse reaction suspected to be due to a drug or other health product as defined in Article L5311- 1 must be reported by the health professional to the vigilance networks (internet Portal for reporting adverse health events) who, after analysis, report them to the ANSM.

11 MEDICO-ECONOMIC STUDY

We will conduct a within-trial cost utility analysis using standard methods, comparing neuropsychologist-supervised online cognitive stimulation to unsupervised open access online cognitive stimulation program from the societal perspective. Outcomes will be reported as quality-adjusted life years (QALYs) and cumulative costs, undiscounted due to the 1-year time horizon (3 months intervention + 9 months follow up).
Costs: We will estimate costs for all healthcare use in both arms, including:

-labor costs for the supervised cognitive stimulation;
-physician services;
-hospitalizations, emergency visits;
-outpatient diagnostic tests;
-drugs, including the use of psychotropic drugs;
-home care;
-health-related out-of-pocket costs
-productivity costs.

Valuation will be conducted using national costs for hospital admissions, the social health insurance for services and purchase prices for drugs. Productivity losses will be valued by the mean per capita GDP.

The table below summarizes resource data collection and valuation

Costs	Source of resource use	Valuation	Point of View	Notes
**Medical costs**				
Hospitalisations & outpatients visits post discharge	eCRF DRG* & length of stay adverse events information	DRG - French national cost study (ENC)	Production costs to the hospital	
		DRG using average reimbursement by the national health insurance*	Payer - national health insurance	
		DRG using average amount not reimbursed by the national health insurance*	Payer - patient out of pocket and private health insurance	
Consultations and medication in ambulatory care - Out of hospital care post discharge: only in relation with the follow up of the cognitive complaints	eCRF prescription information not in study centre collected from patient	Operative public sector 1 tariffs for physicians using the Nomenclature Generale des actes professionnels and the Ameli/Assurance Maladie reimbursement tariffs for the type of physician as recorded in the eCRF and pharmacy prices	Payer - national health insurance	Reimbursement rates at time of data analysis
			Payer - patient out of pocket and private health insurance	
Emergency room visits to centre not leading to hospitalisations	eCRF adverse events information	Cours des Comptes average cost estimate of a visit	Production costs to the hospital	Emergency visits that lead to hospitalisations are included in the DRG for the hospitalisation
		Flat fee	Payer - national health insurance	
		Flat fee	Payer - patient out of pocket and private health insurance	
**Non medical costs**				

Work absences	eCRF	Average daily rate for work absence	Payer - national health insurance	Average daily allowance
		Daily rate to subsidise absence (private sector)	Payer - employer	Difference between average daily sickness benefit and average daily wage
		Loss of income	Payer - patient	
Complementary and alternative therapies ex. acupuncture, homeopathy, sophrology, pychology		Price paid	Payer - patient	

* Diagnosis-Related Groups (DRG)

The costs of healthcare are compared between the supervised and unsupervised arms for the period based on healthcare use collected prospectively at the patient level using the eCRF, hospitals' claims data.
Overall quality of life will be assessed using the EQ-5D-5L, which provides a descriptive profile and a single index value for health status. The questionnaire will be filled at each follow up visit. The measure will be used for the economic evaluation, to compute QALYs.
Cumulative costs and QALYs for each trial arm will be estimated and compared in order to calculate the incremental cost utility ratio. The results will be expressed as the incremental cost per QALYs (based on the EQ-5D). We will evaluate uncertainty and estimate confidence intervals around the estimates, using both deterministic and probabilistic methods. We will account for correlation between costs and health outcomes using appropriate bivariate methods. Cost-effectiveness acceptability curves will be used to graphically represent the probability that the intervention would be cost-effective for cost-effectiveness thresholds of €50 000 and €100 000 per QALY gained (multiple thresholds for sensitivity analyses). Reporting will follow the Consolidated Health Economic Evaluation Reporting Standards statement.

12 STATISTICAL CONSIDERATIONS

12.1 SAMPLE SIZE

In our previous COG-Reduc trial (NCT01788618) conducted among 168 patients randomized in 3 groups (56 patients per group), we have observed a difference of 5.2 points (16.3, Standard Deviation SD 14.7 vs 11.1, SD 14.8) on the FACT-Cog PCI in favour of the software-based cognitive stimulation program supervised by a neuropsychologist as compared to a home-based unsupervised cognitive stimulation exercises using a dedicated booklet (Dos Santos et al., 2020).

Given that the present proposal aims to assess the added value of remotely supervision by a neuropsychologist of an online cognitive stimulation, as compared to an online cognitive stimulation without any supervision, we similarly expect a mean difference of score change between end of program (at 12 weeks) and baseline of at least 5.2 points in the continuous FACT-Cog PCI between experimental and control arms.

As suggested previously (Bell et al., 2018), such difference is clinically important. Using a two sample t-test with equal variances (allocation-ratio 1:1, bilateral alpha risk 0.05, power 80%) and assuming a standard deviation of 14.7, 127 assessable patients per arm are required. To anticipate 15% of non-assessable patients, we plan to enrol 300 patients (150/arm).

12.2 STATISTICAL ANALYSIS

All analyses will be described in a detailed statistical analyses plan (SAP), and will be performed using the intent-to-treat (ITT) population. Any deviation to SAP and protocol changes that may occur after study initiation will appear and discussed in the final report of the study.

12.2.1 Primary objective

The continuous FACT-Cog PCI subscale difference between the end of the 12-week intervention and baseline measures will be computed and compared between experimental and control groups by using a bilateral Student test.

12.2.2 Secondary objectives

Self-reported outcomes (quality of life questionnaires) and objective cognitive evaluations will be measured by scores and described as follows:

- Quantitative variables will be summarized using quartiles and range (mean and standard deviation for Gaussian-like variables).
- Categorical variables will be summarized using numbers and proportions.

To measure evolution through time, comparison of scores between two assessments will first be performed by paired T-test (or the Wilcoxon signed-rank test for paired data for non-gaussian variables) in each group. Then, comparison of cognitive evolution between groups will employ a linear mixed model with random patient effect, adjusted on age, composite criteria of fatigue/anxiety/depression, current physical activity (stratification criteria).

For all statistical analysis, two-sided p-values <0.05 will be considered statistically significant.

13 QUALITY CONTROL

13.1 TRAINING OF PARTICIPATING TEAMS

To ensure consistency among all participating centres, a comprehensive manual of procedures will be prepared and approved by each centre prior to the start of the study. The study staff at each center will be trained on the consent process, eligibility screening, on administering the MoCA test (including the prerequired formation for non-neuropsychologists, planned in the trial budget), the questionnaires, assisting patients using the CNS vital Sign battery, and using the HappyNeuron PRESCO platform.

13.2 NEUROPSYCHOLOGICAL SUPERVISION

To ensure consistent supervision, the neuropsychologist coordinator will thoroughly review a semi-structured interview questionnaire for the initial and follow-ups interviews of patients of the experimental arm, before the start of study. Regularly weekly meetings will be held to discuss patients' progress and participation in the program as required.

The educational content of these supervision sessions will be developed in advance and standardized among neuropsychologists involved in the project. This content will be based on the educational material already used by the neuropsychologists in the centre for cognitive workshops (Schuurs & Green, 2013).

13.3 PROTOCOL DEVIATIONS

A standard protocol deviations form will be used to track any deviations. The study staff will maintain the protocol deviations log. The study staff will also record deviations discovered in auditing the data and reconcile errors.

13.4 MONITORING

The neuropsychologist coordinator will perform routine monitoring of adherence to the program and patient's improvements. The study staff will monitor data collection and for protocol deviations.

14 ETHICS AND REGULATORY CONSIDERATIONS

The study will be conducted in accordance with the French Public Health Law, specially relating to research involving the biomedical human person of the Public Health Code, articles L1121-1 and following (Law No. 2012-300 of 05/03/2012 as amended by Ordinance No. 2016-800 of 16 June 2016), the Bioethics Law, the law related to the protection of physical persons for the treatments of personal data and related to information technology, database and liberties, the Helsinki declaration and the Good Clinical Practices.

14.1 CLINICAL TRIAL AUTHORISATION

An authorization request will be sent by the sponsor to the French regulatory authorities before the study initiation:

- Ethics Committee (Committee for the Protection of Persons, CPP)

An Information will be given to the Competent Authority (ANSM), with transmission of the synopsis of the study and the favorable opinion of the CPP.

This study is under of the "Reference Methodology" (MR-001) in application of the provisions of article 54 paragraph 5 of the law of 6 January 1978 as amended relating to data processing, files and freedoms. This change was approved by decision of 5 January 2006.

The François Baclesse Center respects the regulations in force, in particular the rights of the persons being treated according to the EU regulation 2016/679 on the protection of data ("RGPD").

Any substantial modification in the protocol about objectives, design, population, evaluation, significant administrative modifications will need the coordinator approval, the sponsor approval, CPP approval and the competent authority information.

14.2 INFORMATION OF PATIENTS INVOLVED IN THE RESEARCH

Patients will be completely and faithfully informed with understandable words on the objectives and constraints of the research, potential risks, required measures for monitoring and safety, of their right to decline the participation in the study or the possibility to withdraw from the study at any time.

All these information are included in the informed consents form given to the patient: one for the main study, other for ancillary studies. The investigator, or the physician who represents him, will collect the signed written informed consent(s) before the definitive inclusion in the study. A copy of the information and consent form signed by the two parties will be given to the patient; the investigator will keep the second copy.

For any significant modification of the protocol related to the objectives of the research, its design, the population, the exams or significant administrative aspects, a new consent from each person participating to the research will be collected if needed.

14.3 INVESTIGATOR RESPONSIBILITIES

The Principal Investigator of each participating center is committed to conduct the clinical trial in accordance with the trial protocol and with the regulations in force, notably the decision of 24 November 2006 related to Good Clinical Practices.

The Principal Investigator is responsible for:

- ✓ Giving to Sponsor his/her curriculum vitae and that of co-investigators
- ✓ Identifying persons involved in the research in his/her team and defining their responsibilities
- ✓ Initiating the inclusion of patients after Sponsor authorization
- ✓ Making the maximum effort to include the required number of patients within the established recruitment period.

Each Investigator is responsible for:

- ✓ Obtaining the signed and dated informed consent and personally signing this consent for each participating patient before any procedure specific to the trial
- ✓ Regularly completing the Case Report Form (CRF) for each patient included in the trial and to allow to CRAs mandated by the Sponsor a direct access to source data in order to validate the data entered in the CRF
- ✓ Dating, correcting and signing any correction in the CRFs and data clarification forms (DCF)
- ✓ Accepting the regular monitoring visits of the monitor and eventual auditors mandated by the Sponsor or inspectors of supervisory authorities

Source documents, defined as any document or original item that allow to prove the existence or accuracy of a data or a fact recorded during the study, will be kept during 15 years by the investigator or the hospital if the source is a hospital medical record.

The archiving of the data will be the responsibility of the investigator and according to the legislation. The patient should keep the data and a patient identification list for a minimum of 15 years after the end of the study.

14.4 DATA CONFIDENTIALITY

The investigator will ensure the confidentiality of all information concerning the project for himself and for all persons involved in the conduct of the trial until the publication of the test results. This confidentiality obligation will not apply to information that the investigator will be required to provide to patients in the context of their participation in the trial or to information already published. The investigator will ensure not to publish, disclose or use, in any way, directly or indirectly, scientific or technical informations of the trial.

The study may not be the subject of any written or oral commentary without the agreement of the sponsor; all the information communicated or obtained during the realization of the test belonging in full right to the sponsor who can freely dispose of it.

15 DATA AND DOCUMENTS KEEPING

15.1 DATA ENTRY AND HANDLING

Data management will be performed by the Data Processing Center (CTD) of the North West Cancéropôle (Centre de Traitement des Données du Cancéropôle Nord-Ouest). The CTD provides a database management software dedicated to clinical research: Ennov Clinical (version 7.5.10, ENNOV / CLINSIGHT, 33155 Cenon, France).

This software package, which is based on an Oracle database architecture, is designed for the overall management of clinical and epidemiological studies, meets the regulatory requirements related to this type of study. The CTD Ennov Clinical instance is validated in its computing environment. A data validation plan will be developed jointly by the Clinical Research Unit and the Data Processing Center and will describe in detail the controls to be performed for each variable.

A database specific to the study will be created, tested and validated before the start of the study. All information required by the protocol must be recorded on the paper observation books - or on the electronic observation booklet - under the responsibility of the principal investigator and an explanation must be provided for each missing data item. The data will have to be entered in these notebooks as they are obtained, and the sponsor will take over the monitoring.

The data will then be checked by the CTD in accordance with the data validation plan.

The database will be frozen after final quality control and then exported to the adequate format for statistical analysis according to an automated and validated procedure.

15.2 ARCHIVING

The sponsor must ensure the archiving of essential documents on the conduct of the study in conditions ensuring their safety, for the minimum duration provided by BPC, 15 years after the end of the research.

These documents are the protocol and annexes including any amendments, original signed information forms and consents, questionnaires, case report forms, follow-up documents, statistical analyzes, the final report of the study.

15.3 PUBLICATION POLICY

The results of this study, property of the Sponsor (Centre François Baclesse), will be published under scientific articles. Publications relating to or resulting from this research will be communicated and submitted for review by the study coordinators to all investigators.

The authors include investigators that have included most patients, the biostatistician who has performed the data analysis, the clinical researcher monitor and the participants who provided substantial contribution to the development of the study, the analysis and interpretation of results and / or the writing of the manuscript.

No publication or presentation of the results will be allowed without the agreement of all the parties. Each investigator will be author in the order determined by the number of eligible patients included. No publication or communication will be performed without the agreement of the coordinating investigator and the Sponsor

with the obligation to mention the name of the Sponsor, the organism which financially supported the conduct of the trial.

Some dedicated publications for ancillary studies will be also performed.

This work will be the property of all authors and will be at their disposal for transversal communications and publications.

Publications related to the results of potential ancillary studies need prior approval of the coordinating investigator and methodologist and will be done after the publication of the main study, which should be cited as reference.

16 FUNDING AND INSURANCE

16.1 FUNDING

Any additional costs referred to the Code of Public Health are being negotiated between the CFB and the representative of the institution, taking into account the financial resources available to the CFB in the frame of its public promotion activities.

However, the CFB will ensure the study implementation and supply of the following material (protocol, CRF, investigator file) needed to the conduct of the study.

In the case of equipment or treatments are provided by other partners, the conditions must be specified in the study agreement.

16.2 INSURANCE

The sponsor has subscribed for the duration of the study an insurance covering his own liability and that of any physician involved in the realization of the study. It will also ensure full compensation for the harmful consequences to search for the person undergoing it and assigns, unless evidence against him that the damage is not attributable to its fault or that of any intervener, without that can be opposite the act of a third party or the voluntary withdrawal of the person who had originally agreed to participating to research (Article L 1121-10).

17 REFERENCES

Barak, A., Hen, L., Boniel-Nissim, M., & Shapira, N. (2008). A comprehensive review and a meta-analysis of the effectiveness of internet-based psychotherapeutic interventions. *Journal of Technology in Human Services*, *26*(2–4), 109–160.

Bell, M. L., Dhillon, H. M., Bray, V. J., & Vardy, J. L. (2018). Important differences and meaningful changes for the functional assessment of cancer therapy-cognitive function (FACT-Cog). *Journal of Patient-Reported Outcomes*, *2*, 1–11.

Binarelli, G., Joly, F., Tron, L., Lefevre Arbogast, S., & Lange, M. (2021). Management of Cancer-Related Cognitive Impairment: A Systematic Review of Computerized Cognitive Stimulation and Computerized Physical Activity. *Cancers*, *13*(20), 5161.

Binarelli, G., Lange, M., Santos, M. D., Grellard, J., Lelaidier, A., Tron, L., Arbogast, S. L., Clarisse, B., & Joly, F. (2021). Multimodal Web-Based Intervention for Cancer-Related Cognitive Impairment in Breast Cancer Patients: Cog-Stim Feasibility Study Protocol. *Cancers*, *13*, null. https://doi.org/10.3390/cancers13194868

Bray, V. J., Dhillon, H. M., & Vardy, J. L. (2018). Systematic review of self-reported cognitive function in cancer patients following chemotherapy treatment. *Journal of Cancer Survivorship*, *12*(4), 537–559. https://doi.org/10.1007/s11764-018-0692-x

Brooks, R. (1996). EuroQol: The current state of play. *Health Policy (Amsterdam, Netherlands)*, *37*(1), 53–72. https://doi.org/10.1016/0168-8510(96)00822-6

Cella, D. F., Tulsky, D. S., Gray, G., Sarafian, B., Linn, E., Bonomi, A., Silberman, M., Yellen, S. B., Winicour, P., Brannon, J., & . (1993). The Functional Assessment of Cancer Therapy scale: Development and validation of the general measure. *Journal of Clinical Oncology*, *11*(0732-183X (Print)), 570–579.

Cella, D., Lai, J. S., Chang, C. H., Peterman, A., & Slavin, M. (2002). Fatigue in cancer patients compared with fatigue in the general United States population. *Cancer*, *94*(0008-543X (Linking)), 528–538.

Chan, R. J., McCarthy, A. L., Devenish, J., Sullivan, K. A., & Chan, A. (2015). Systematic review of pharmacologic and non-pharmacologic interventions to manage cognitive alterations after chemotherapy for breast cancer. *European Journal of Cancer*, *51*(4), 437–450.

Cheung, Y. T., Shwe, M., Chui, W. K., Chay, W. Y., Ang, S. F., Dent, R. A., Yap, Y. S., Lo, S. K., Ng, R. C. H., & Chan, A. (2012). Effects of chemotherapy and psychosocial distress on perceived cognitive disturbances in Asian breast cancer patients. *Annals of Pharmacotherapy*, *46*(12), 1645–1655.

Clare, L., & Woods, R. T. (2004). Cognitive training and cognitive rehabilitation for people with early-stage Alzheimer's disease: A review. *Neuropsychological Rehabilitation*, *14*(4), 385–401. https://doi.org/10.1080/09602010443000074

Conklin, H. M., Ogg, R. J., Ashford, J. M., Scoggins, M. A., Zou, P., Clark, K. N., Martin-Elbahesh, K., Hardy, K. K., Merchant, T. E., & Jeha, S. (2015). Computerized cognitive training for amelioration of cognitive late effects among childhood cancer survivors: A randomized controlled trial. *Journal of Clinical Oncology*, *33*(33), 3894.

Craig, C. L., Marshall, A. L., Sjöström, M., Bauman, A. E., Booth, M. L., Ainsworth, B. E., Pratt, M., Ekelund, U., Yngve, A., Sallis, J. F., & Oja, P. (2003). International physical activity questionnaire: 12-country reliability and validity. *Medicine and Science in Sports and Exercise*, *35*(8), 1381–1395. https://doi.org/10.1249/01.MSS.0000078924.61453.FB

Damholdt, M. F., Mehlsen, M., O'Toole, M. S., Andreasen, R. K., Pedersen, A. D., & Zachariae, R. (2016). Web-based cognitive training for breast cancer survivors with cognitive complaints-a randomized controlled trial. *Psychooncology.*, *1099-1611 (Electronic)*.

Dos Santos, M., Hardy-Léger, I., Rigal, O., Licaj, I., Dauchy, S., Levy, C., Noal, S., Segura, C., Delcambre, C., Allouache, D., Parzy, A., Barriere, J., Petit, T., Lange, M., Capel, A., Clarisse, B., Grellard, J. M., Lefel, J., & Joly, F. (2020). Cognitive rehabilitation program to improve cognition of cancer patients treated with chemotherapy: A 3-arm randomized trial. *Cancer*, *126*(24), 5328–5336. https://doi.org/10.1002/cncr.33186

Gehring, K., Sitskoorn, M. M., Gundy, C. M., Sikkes, S. A. M., Klein, M., Postma, T. J., van den Bent, M. J., Beute, G. N., Enting, R. H., Kappelle, A. C., Boogerd, W., Veninga, T., Twijnstra, A., Boerman, D. H., Taphoorn, M. J. B., & Aaronson, N. K. (2009). Cognitive Rehabilitation in Patients With Gliomas: A Randomized, Controlled Trial. *Journal of Clinical Oncology*, *27*(22), 3712–3722. https://doi.org/10.1200/JCO.2008.20.5765

Haberlin, C., O'Dwyer, T., Mockler, D., Moran, J., O'Donnell, D. M., & Broderick, J. (2018). The use of eHealth to promote physical activity in cancer survivors: A systematic review. *Supportive Care in Cancer*, *26*(10), 3323–3336.

Janelsins, M. C., Heckler, C. E., Peppone, L. J., Kamen, C., Mustian, K. M., Mohile, S. G., Magnuson, A., Kleckner, I. R., Guido, J. J., & Young, K. L. (2017). Cognitive complaints in survivors of breast cancer after chemotherapy compared with age-matched controls: An analysis from a nationwide, multicenter, prospective longitudinal study. *Journal of Clinical Oncology*, *35*(5), 506.

Joly, F., Giffard, B., Rigal, O., De Ruiter, M. B., Small, B. J., Dubois, M., LeFel, J., Schagen, S. B., Ahles, T. A., Wefel, J. S., Vardy, J. L., Pancré, V., Lange, M., & Castel, H. (2015). Impact of Cancer and Its Treatments on Cognitive Function: Advances in Research From the Paris International Cognition and Cancer Task Force Symposium and Update Since 2012. *Journal of Pain and Symptom Management*, *50*(6), 830–841. https://doi.org/10.1016/j.jpainsymman.2015.06.019

Joly, F., Lange, M., Rigal, O., Correia, H., Giffard, B., Beaumont, J. L., Clisant, S., & Wagner, L. (2012). French version of the Functional Assessment of Cancer Therapy-Cognitive Function (FACT-Cog) version 3. *Supportive Care in Cancer: Official Journal of the Multinational Association of Supportive Care in Cancer*, *20*(12), 3297–3305. https://doi.org/10.1007/s00520-012-1439-2

Kim, Y., & Kang, S. J. (2019). Computerized programs for cancer survivors with cognitive problems: A systematic review. *Journal of Cancer Survivorship*, *13*(6), 911–920. https://doi.org/10.1007/s11764-019-00807-4

Koppelmans, V., Breteler, M., Boogerd, W., Seynaeve, C., Gundy, C., & Schagen, S. (2012). Neuropsychological performance in survivors of breast cancer more than 20 years after adjuvant chemotherapy. *Journal of Clinical Oncology*, *30*(10), 1080–1086.

Lange, M., Joly, F., Vardy, J., Ahles, T., Dubois, M., Tron, L., Winocur, G., De Ruiter, M., & Castel, H. (2019). Cancer-related cognitive impairment: An update on state of the art, detection, and management strategies in cancer survivors. *Annals of Oncology*, *30*(12), 1925–1940.

Lange, M., Licaj, I., Clarisse, B., Humbert, X., Grellard, J.-M., Tron, L., & Joly, F. (2019). Cognitive complaints in cancer survivors and expectations for support: Results from a web-based survey. *Cancer Medicine*, *8*(5), 2654–2663. https://doi.org/10.1002/cam4.2069

Lonkhuizen, P. J. C., Klaver, K. M., Wefel, J. S., Sitskoorn, M. M., Schagen, S. B., & Gehring, K. (2019). Interventions for cognitive problems in adults with brain cancer: A narrative review. *European Journal of Cancer Care*, *28*(3). https://doi.org/10.1111/ecc.13088

Luxton, D. D. (2015). *Artificial Intelligence in Behavioral and Mental Health Care*. Academic Press.

Marks, I. M., Cavanagh, K., & Gega, L. (2007). *Hands-on help: Computer-aided psychotherapy*. Psychology Press.

Mihuta, M. E., Green, H. J., & Shum, D. H. K. (2018). Web-based cognitive rehabilitation for survivors of adult cancer: A randomised controlled trial. *Psycho-Oncology*, *27*(4), 1172–1179. https://doi.org/10.1002/pon.4615

Morin, C. M., Belleville, G., Bélanger, L., & Ivers, H. (2011). The Insomnia Severity Index: Psychometric Indicators to Detect Insomnia Cases and Evaluate Treatment Response. *Sleep*, *34*(5), 601–608.

Munir, F., Burrows, J., Yarker, J., Kalawsky, K., & Bains, M. (2010). Women's perceptions of chemotherapy-induced cognitive side affects on work ability: A focus group study. *Journal of Clinical Nursing*, *19*(9–10), 1362–1370. https://doi.org/10.1111/j.1365-2702.2009.03006.x

Murray, E., Burns, J., Tai, S. S., Lai, R., & Nazareth, I. (2004). Interactive Health Communication Applications for people with chronic disease. *Cochrane Database of Systematic Reviews*, *4*.

Myers, J. S. (2013). Cancer- and Chemotherapy-Related Cognitive Changes: The Patient Experience. *Seminars in Oncology Nursing*, *29*(4), 300–307. https://doi.org/10.1016/j.soncn.2013.08.010

Nieuwenhuijsen, K., Boer, A. de, Spelten, E., Sprangers, M. A. G., & Verbeek, J. H. A. M. (2009). The role of neuropsychological functioning in cancer survivors' return to work one year after diagnosis. *Psycho-Oncology*, *18*(6), 589–597. https://doi.org/10.1002/pon.1439

Padgett, L. S., Van Dyk, K., Kelly, N. C., Newman, R., Hite, S., & Asher, A. (2020). Addressing Cancer-Related Cognitive Impairment in Cancer Survivorship. *Oncology Issues*, *35*(1), 52–57. https://doi.org/10.1080/10463356.2020.1692601

Schuurs, A., & Green, H. J. (2013). A feasibility study of group cognitive rehabilitation for cancer survivors: Enhancing cognitive function and quality of life. *Psycho-Oncology*, *22*(5), 1043–1049. https://doi.org/10.1002/pon.3102

Smidt, K., Mackenzie, L., Dhillon, H., Vardy, J., Lewis, J., & Loh, S. Y. (2016). The perceptions of Australian oncologists about cognitive changes in cancer survivors. *Supportive Care in Cancer*, *24*(11), 4679–4687. https://doi.org/10.1007/s00520-016-3315-y

Triberti, S., Savioni, L., Sebri, V., & Pravettoni, G. (2019). eHealth for improving quality of life in breast cancer patients: A systematic review. *Cancer Treatment Reviews*, *74*, 1–14. https://doi.org/10.1016/j.ctrv.2019.01.003

Von Ah, D., Habermann, B., Carpenter, J. S., & Schneider, B. L. (2013). Impact of perceived cognitive impairment in breast cancer survivors. *European Journal of Oncology Nursing*, *17*(2), 236–241.

Wagner, L. I., Sweet, J., Butt, Z., Lai, J. S., & Cella, D. (2009). Measuring Patient Self-Reported Cognitive Function: Development of the Functional Assessment of Cancer Therapy-Cognitive Function Instrument. *The Journal of Supportive Oncology*, *7*, W32–W39.

Yamada, T. H., Denburg, N. L., Beglinger, L. J., & Schultz, S. K. (2010). Neuropsychological outcomes of older breast cancer survivors: Cognitive features ten or more years after chemotherapy. *The Journal of Neuropsychiatry and Clinical Neurosciences*, *22*(1), 48–54.

Zigmond, A. S., & Snaith, R. P. (1983). The hospital anxiety and depression scale. *Acta Psychiatr Scand.*, *67*(0001-690X (Print)), 361–370.
