# Supplementary material for: Remotely supervised online cognitive training to reduce cognitive difficulties following chemotherapy in patients treated for localized breast cancer: Protocol of the Cog-Stim2 multicenter randomized controlled trial
Source: PLoS One. 2025 Nov 13;20(11):e0335124. doi: 10.1371/journal.pone.0335124 (PMC12614541; doi:10.1371/journal.pone.0335124)

**REGULATORY AUTHORIZATIONS for implementation of the Cog-Stim2 Trial**

The Cog-Stim2 trial has received ethical approval from the Comité de Protection des Personnes Nord Ouest III in August 2023 (N° ID RCB: 2023-A01134-41, Réf. SI RIPH 2G: 23.01946.000193).

**Initial ethical approval dated from August 16, 2023**
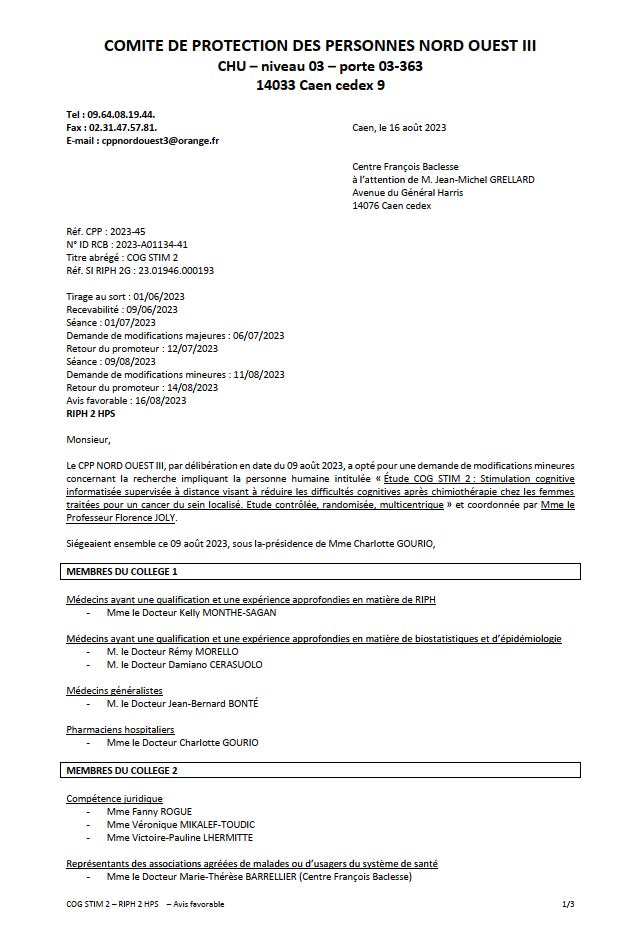

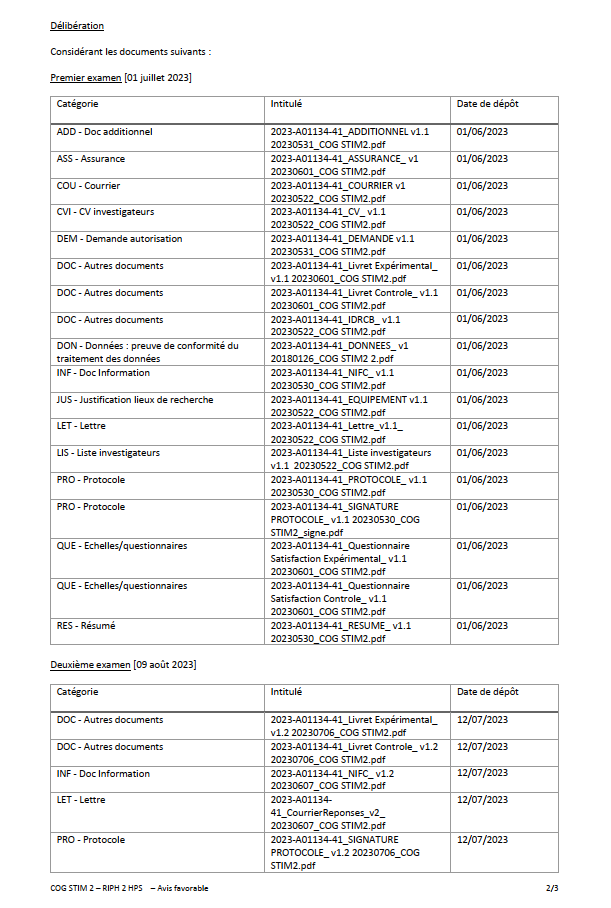

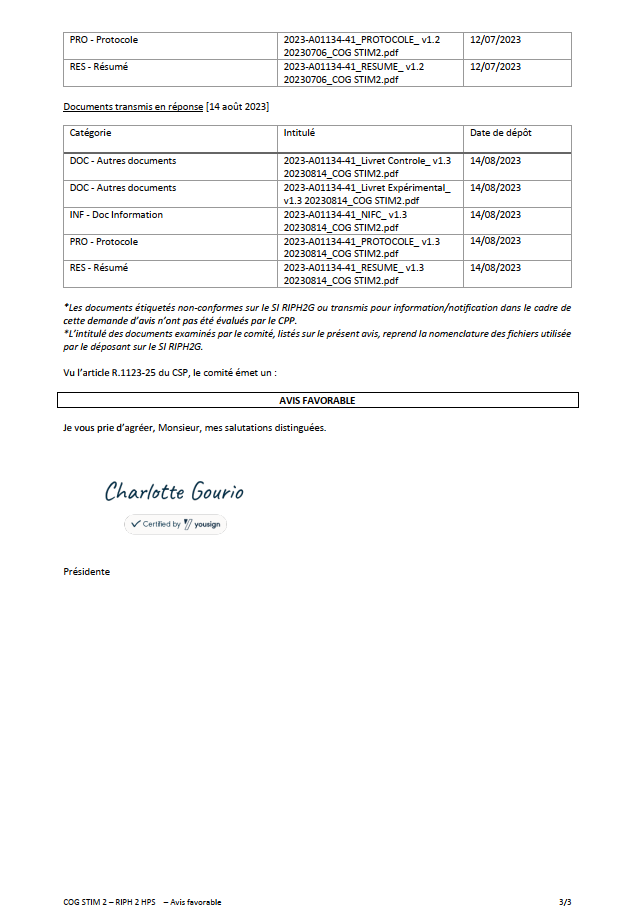


**NORTHWEST PERSONAL PROTECTION COMMITTEE III**

**CHU – Niveau 03 - Porte 03-508 - Avenue de la Côte de Nacre**

**14033 Caen Cedex 09**

Tel. : 09.64.08.19.44

Fax: 02.31.47.57.81

E-mail: cppnordouest3@orange.fr

Caen, August 16, 2023

Centre François Baclesse

TO the attention of Jean-Michel GRELLARD

Avenue du général Harris

14076 Caen cedex 5

Réf. CPP : 2023-45

N° ID RCB : 2023-A01134-41

Short Title : COG STIM 2

Réf. SI RIPH 2G : 23.01946.000193

Randomization: 01/JUNE/2023

Recevability: 09/JUNE/2023

Session: 01/JULY/2023

Request for major modifications: 06/JULY/2023

Sponsor response: 12/JULY/2023

Session: 09/AUGUST/2023

Request for minor modifications: 11/AUGUST/2023

Sponsor response: 14/AUGUST/2023

Favorable opinion: 16/AUGUST/2023

RIPH 2 HPS

Sir,

The Northwest Personal protection Committee III, by deliberation dated from August 9, 2023, issued a request for minor modification concerning the study on human persons entitled “COG STIM2 study : Remotely supervised online cognitive training to reduce cognitive difficulties following chemotherapy in patients treated for localized breast cancer: a multicenter randomized controlled trial” and led by Prof Florence JOLY [Caen].

Sitting together this August 9, 2023, under the chairmanship of Mrs. Charlotte GOURIO,

Members of the first college

Physicians with extensive qualifications and experience in studies involving human person (RIPH)

- Dr. Kelly MONTHE-SAGAN

Physicians with extensive qualifications and experience in biostatistics and epidemiology

- Dr. Rémy MORELLO

- Dr. Damiano CERASUOLO

General Practitioners

- Dr. Jean-Bernard BONTÉ

Hospital Pharmacists

- Dr. Charlotte GOURIO

Members of the second college

Legal Expertise

- Ms. Fanny ROGUE

- Ms. Véronique MIKALEF-TOUDIC

- Ms. Victoire-Pauline LHERMITTE

Representatives of approved patient and healthcare system user associations

- Dr. Marie-Thérèse BARRELLIER (François Baclesse Center)

Deliberation

Considering the following documents:

First examination (01 July 2023)

| Category | Name | Date of submission |
| --- | --- | --- |
| DD – Additional File | 2023-A01134-41_ADDITIONNEL v1.1 20230531_COG STIM2.pdf | 01/JUNE/2023 |
| ASS - Insurance | 2023-A01134-41_ASSURANCE_ v1 20230601_COG STIM2.pdf | 01/JUNE/2023 |
| COU - Mail | 2023-A01134-41_COURRIER v1 20230522_COG STIM2.pdf | 01/JUNE/2023 |
| CVI - CV investigators | 2023-A01134-41_CV_ v1.1 20230522_COG STIM2.pdf | 01/JUNE/2023 |
| DEM – Request for autorization | 2023-A01134-41_DEMANDE v1.1 20230531_COG STIM2.pdf | 01/JUNE/2023 |
| DOC - Other documents | 2023-A01134-41_Livret Expérimental_ v1.1 20230601_COG STIM2.pdf | 01/JUNE/2023 |
| DOC - Other documents | 2023-A01134-41_Livret Controle_ v1.1 20230601_COG STIM2.pdf | 01/JUNE/2023 |
| DOC - Other documents | 2023-A01134-41_IDRCB_ v1.1 20230522_COG STIM2.pdf | 01/JUNE/2023 |
| DON - Data: proof of compliance of data processing | 2023-A01134-41_DONNEES_ v1 20180126_COG STIM2 2.pdf | 01/JUNE/2023 |
| INF - Information file | 2023-A01134-41_NIFC_ v1.1 20230530_COG STIM2.pdf | 01/JUNE/2023 |
| JUS - Justification of research locations | 2023-A01134-41_EQUIPEMENT v1.1 20230522_COG STIM2.pdf | 01/JUNE/2023 |
| LET – Letter | 2023-A01134-41_Lettre_v1.1_ 20230522_COG STIM2.pdf | 01/JUNE/2023 |
| LIS – Investigators Listing | 2023-A01134-41_Liste investigateurs v1.1 20230522_COG STIM2.pdf | 01/JUNE/2023 |
| PRO - Protocol | 2023-A01134-41_PROTOCOLE_ v1.1 20230530_COG STIM2.pdf | 01/JUNE/2023 |
| PRO - Protocole | 2023-A01134-41_SIGNATURE PROTOCOLE_ v1.1 20230530_COG STIM2_signe.pdf | 01/JUNE/2023 |
| QUE - Scales/questionnaires | 2023-A01134-41_Questionnaire Satisfaction Expérimental_ v1.1 20230601_COG STIM2.pdf | 01/JUNE/2023 |
| QUE - Scales/questionnaires | 2023-A01134-41_Questionnaire Satisfaction Controle_ v1.1 20230601_COG STIM2.pdf | 01/JUNE/2023 |
| RES - Synopsis | 2023-A01134-41_RESUME_ v1.1 20230530_COG STIM2.pdf | 01/JUNE/2023 |

Second examination (09 August 2023)

| Category | Name | Date of submission |
| --- | --- | --- |

| DOC - Other documents | 2023-A01134-41_Livret Expérimental_ v1.2 20230706_COG STIM2.pdf | 12/JULY/2023 |
| --- | --- | --- |
| DOC - Other documents | 2023-A01134-41_Livret Controle_ v1.2 20230706_COG STIM2.pdf | 12/JULY/2023 |
| INF - Information File | 2023-A01134-41_NIFC_ v1.2 20230607_COG STIM2.pdf | 12/JULY/2023 |
| LET – Letter | 2023-A01134- 41_CourrierReponses_v2_ 20230607_COG STIM2.pdf | 12/JULY/2023 |
| PRO - Protocol | 2023-A01134-41_SIGNATURE PROTOCOLE_ v1.2 20230706_COG STIM2.pdf | 12/JULY/2023 |
| PRO – Protocol | 2023-A01134-41_PROTOCOLE_ v1.2 20230706_COG STIM2.pdf | 12/JULY/2023 |
| RES – Synopsis | 2023-A01134-41_RESUME_ v1.2 20230706_COG STIM2.pdf | 12/JULY/2023 |

Files submitted in response [14 August 2023]

| Category | Name | Date of submission |
| --- | --- | --- |

| DOC - Other documents | 2023-A01134-41_Livret Controle_ v1.3 20230814_COG STIM2.pdf | 14/AUGUST/2023 |
| --- | --- | --- |
| DOC - Other documents | 2023-A01134-41_Livret Expérimental_ v1.3 20230814_COG STIM2.pdf | 14/AUGUST/2023 |
| INF - Information File | 2023-A01134-41_NIFC_ v1.3 20230814_COG STIM2.pdf | 14/AUGUST/2023 |
| PRO – Protocol | 2023-A01134-41_PROTOCOLE_ v1.3 20230814_COG STIM2.pdf | 14/AUGUST/2023 |
| RES - Synopsis | 2023-A01134-41_RESUME_ v1.3 20230814_COG STIM2.pdf | 14/AUGUST/2023 |

*Documents labeled non-compliant on the RIPH2G IS or submitted for information/notification as part of this request for an opinion have not been evaluated by the Ethics Committee.

*The titles of the documents reviewed by the committee, listed in this opinion, reflect the file nomenclature used by the submitter on the RIPH2G IS.

In view of article R.1123-25 of the Public Health Code, the Northwest Personal protection Committee III therefore opted for:

| **FAVORABLE OPINION** |
| --- |

Please accept, Sir, my best regards


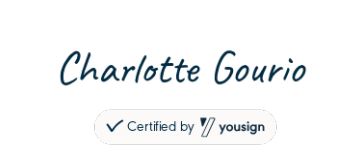


Chairwoman

**FUNDINGS**

This trial (NCT06027632) is granted by the French Cancer Institute and French Health Ministry (PHRC-K 22-038, available on [Les projets retenus - Ministère du Travail, de la Santé, des Solidarités et des Familles](https://sante.gouv.fr/systeme-de-sante/innovation-et-recherche/l-innovation-et-la-recherche-clinique/appels-a-projets/article/les-projets-retenus), file ‘Projets retenus au PHRC-K en 2021’: phrck_21_resultatsselection_v0-3_20230703_mel’ – Selected projects to PHRC-K in 2021).


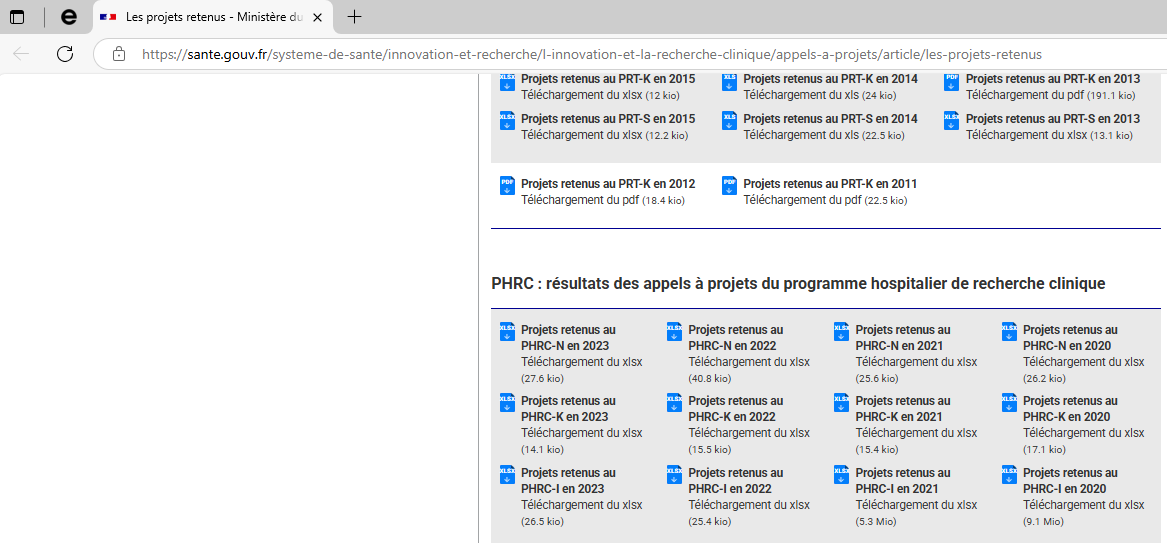


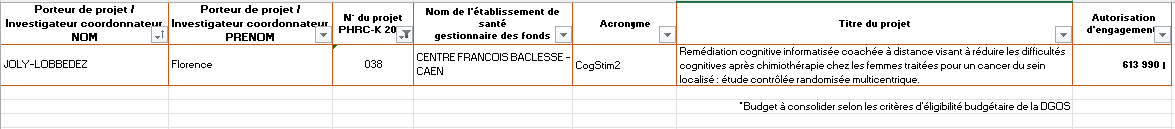

Supplement: S3 File — (DOCX) [file pone.0335124.s003.docx]
