# Supplementary material for: Remotely supervised online cognitive training to reduce cognitive difficulties following chemotherapy in patients treated for localized breast cancer: Protocol of the Cog-Stim2 multicenter randomized controlled trial
Source: PLoS One. 2025 Nov 13;20(11):e0335124. doi: 10.1371/journal.pone.0335124 (PMC12614541; doi:10.1371/journal.pone.0335124)

**FUNDINGS**

This trial (NCT06027632) is granted by the French Cancer Institute and French Health Ministry (PHRC-K 22-038, available on [Les projets retenus - Ministère du Travail, de la Santé, des Solidarités et des Familles](https://sante.gouv.fr/systeme-de-sante/innovation-et-recherche/l-innovation-et-la-recherche-clinique/appels-a-projets/article/les-projets-retenus), file ‘Projets retenus au PHRC-K en 2021’: phrck_21_resultatsselection_v0-3_20230703_mel’ – Selected projects to PHRC-K in 2021).


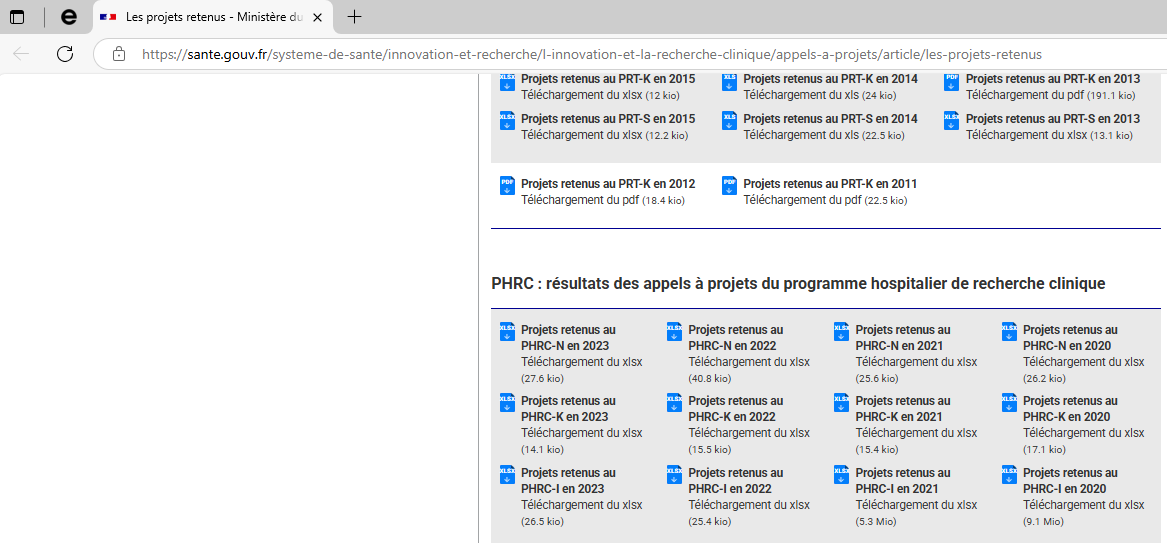


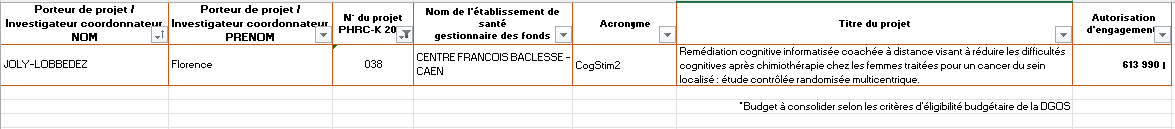

Supplement: S4 File — (DOCX) [file pone.0335124.s004.docx]
